# Conversion of 2D MXene to Multi‐Low‐Dimensional GerMXene Superlattice Heterostructure

**DOI:** 10.1002/adfm.202108495

**Published:** 2021-11-30

**Authors:** Alireza Rafieerad, Ahmad Amiri, Weiang Yan, Hossein Eshghi, Sanjiv Dhingra

**Affiliations:** ^1^ Regenerative Medicine Program Department of Physiology and Pathophysiology Rady Faculty of Health Sciences Institute of Cardiovascular Sciences St. Boniface Hospital Albrechtsen Research Centre University of Manitoba Winnipeg Manitoba R2H2A6 Canada; ^2^ J. Mike Walker ‘66 Mechanical Engineering Department Texas A&M University College Station TX 77843 USA; ^3^ Department of Chemistry Faculty of Science Ferdowsi University of Mashhad Mashhad 91775‐1436 Iran

**Keywords:** conversion model, germanane quantum dots, GerMXene, multidimensional heterostructure, titanium germanide

## Abstract

Integration of 2D structures into other low‐dimensional materials results in the development of distinct van der Waals heterostructures (vdWHSs) with enhanced properties. However, obtaining 2D–1D–0D vdWHSs of technologically useful next generation materials, transition‐metal carbide MXene and monoelemental Xene nanosheets in a single superlattice heterostructure is still challenging. Here, the fabrication of a new multidimensional superlattice heterostructure “GerMXene” from exfoliated M_3_X_2_T*_x_* MXene and hydrogenated germanane (GeH) crystals, is reported. Direct experimental evidence for conversion of hydrothermally activated titanium carbide MXene (A‐MXene) to GerMXene heterostructure through the rapid and spontaneous formation of titanium germanide (TiGe_2_ and Ti_6_Ge_5_) bonds, is provided. The obtained GerMXene heterostructure possesses enhanced surface properties, aqueous dispersibility, and Dirac signature of embedded GeH nanosheets as well as quantum dots. GerMXene exhibits functional bioactivity, electrical conductivity, and negative surface charge, paving ways for its applications in biomedical field, electronics, and energy storage.

## Graphical abstract

Synthesis of new multidimensional superlattice heterostructure “GerMXene” from MXene and Xene nanosheets is reported. This protocol demonstrates the conversion of 2D transition‐metal carbide MXene to a quantum‐manipulated heterostructure with unique structural and material properties. GerMXene crystals possess high surface activity and electro‐conductivity, suggesting its potential applications in multiple fields. Furthermore, GerMXene displays excellent bioactivity for tissue engineering and regenerative medicine.

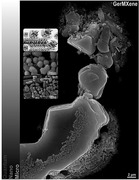

## 1 Introduction

Recently, there has been growing interest in the development of van der Waals heterostructures (vdWHSs) for applications in biomedical field, electronics and energy storage.^[^ ^1^, ^2^ ^]^ The strategies to synthesize vdWHSs have not been limited to the 2D nanosheets rather there have been significant efforts to tailor the microstructure and properties of different materials.^[^ ^3^, ^4^, ^5^, ^6^ ^]^ In fact, several 2D–0D, 2D–1D, and 2D–3D vdWHSs are obtained through chemical interaction of vertical 2D materials with other materials of different dimensionality.^[^ ^7^, ^8^ ^]^ As a result, the obtained mixed‐dimensional vdWHSs provides enhanced surface and material properties to these heterostructures compared to their parent materials.^[^ ^1^, ^9^, ^10^, ^11^, ^12^ ^]^

Ever since the discovery of 2D transition metal carbides “MXene” in 2011, interdisciplinary attention has been paid to the structural and electronic properties of these materials.^[^ ^13^, ^14^, ^15^, ^16^ ^]^ The bulked MXene nanosheets (M*_n_*_+1_X*_n_* T*_x_*, *n* = 1–3) which are synthesized by selective etching of their MAX phases, have high electron density at Fermi level with unique physicochemical, electrical and biological properties.^[^ ^17^, ^18^, ^19^, ^20^, ^21^, ^22^, ^23^ ^]^ However, increasing concerns related to potential phase‐transformation of MXene nanosheets in aqueous dispersions under ambient conditions cannot be ignored.^[^ ^24^, ^25^, ^26^ ^]^ Additionally, van der Waals forces available between MXene flakes tend to restack and aggregate the layers, hindering its long‐term applications.^[^ ^27^ ^]^ These concerns have significantly limited the growth in the field. Therefore, new strategies to synthesize multidimensional MXene superlattice vdWHSs are urgently needed. Furthermore, the recent development of atomically thin monoelemental 2D materials (Xenes) has expanded the capacity in the field.^[^ ^28^, ^29^, ^30^, ^31^, ^32^, ^33^ ^]^ These crystalline materials include one of the metalloids of groups 13–16 in their structures and have gained strength due to unique structural and electronic properties. Additionally, the intrinsic passivation of Xenes gives them the capability to stack low‐dimensional materials and construct hybrid or heterostructures. In particular, germanane (GeH) is one of the recently synthesized 2D monoelemental crystals.^[^ ^34^, ^35^, ^36^ ^]^ GeH is a hydrogen‐terminated multilayer allotrope of germanium and is shown to possess high electron mobility, direct bandgap, active optical properties and quantum spin Hall effect in kagome lattice.^[^ ^31^, ^37^, ^38^ ^]^ GeH also offers considerable electrical conductivity in layered van der Waals materials.^[^ ^34^, ^39^ ^]^ However, the colloidal dispersibility of GeH nanosheets in aqueous media needs to be significantly improved while retaining its desirable properties.^[^ ^30^ ^]^

**Figure 1:**
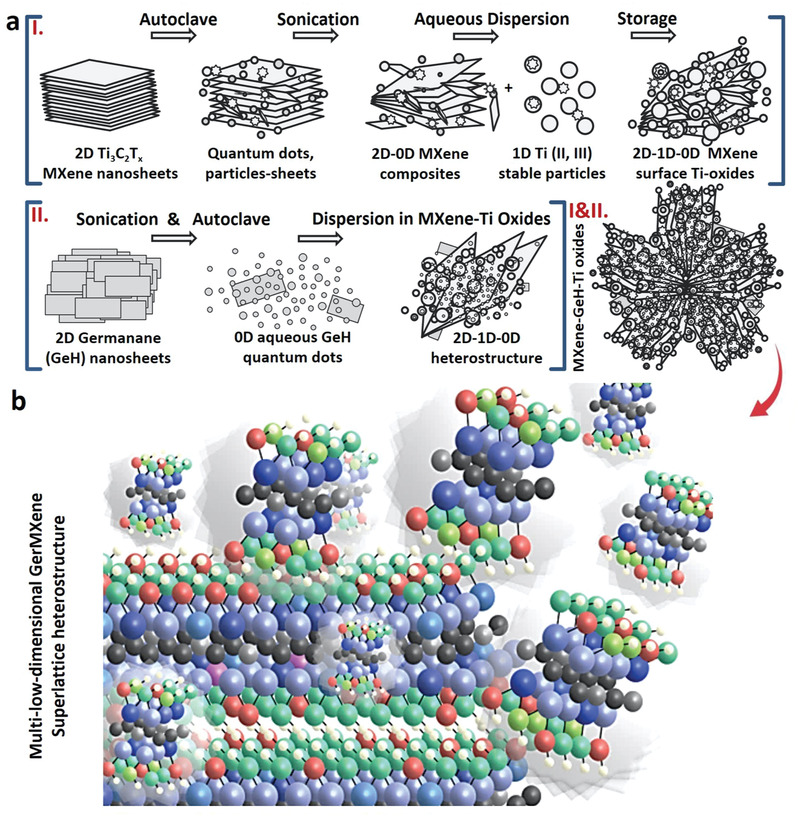
Schematic illustration of the synthesis of GerMXene superlattice heterostructure. a) Schematic model showing the conversion of 2D titanium carbide MXene (Ti_3_C_2_T*_x_*) nanosheets to multilow‐dimensional activated MXene (A‐MXene) crystals. A‐MXene material includes the microstructural characteristics of Ti_3_C_2_T*_x_* nanosheets, quantum dots as well as stable surface titanium oxides. The synthesis of hydrogenated 2D germanane (GeH) from germanium bulks and subsequent conversion to 0D GeH quantum dots. b) Illustration of synthesis model of quantum manipulation and functionalization of M_3_X_2_T*_x_* MXene nanosheets to form a new multilow‐dimensional superlattice GerMXene heterostructure. In the picture black, gray, pink, beige, light blue and dark blue represent carbon, titanium, aluminum, hydrogen, the lowest and the highest germanium atoms, respectively. Furthermore, the functional groups available on the surface of GerMXene are coded in red and green.

The fabrication of 2D vdWHSs with distinct geometries has been reported for graphene, boron nitride and transition‐metal dichalcogenides.^[^ ^1^, ^3^, ^4^, ^5^, ^10^, ^11^, ^12^, ^40^, ^41^ ^]^ However, development of multidimensional superlattice vdWHSs of wonder materials‐ MXenes and Xenes has not been explored yet. The current study reports a versatile strategy to synthesize a unique heterostructure “GerMXene” using 2D MXene and GeH materials. We have employed autoclave treatment method for conversion of Ti_3_C_2_T*_x_* MXene nanosheets to a multilow‐dimensional superlattice material. The uniqueness of this study resides in two important findings; first is activation of 2D Ti_3_C_2_T*_x_* nanosheets to form activated MXene (A‐MXene) composite displaying the morphology and specific characteristics of MXene nanosheets, quantum dots as well as stable surface titanium oxide nanoparticles in a single material. This has not been reported so far for any MXene in literature. Second, the embedding of GeH derived quantum dots in the atomic structure of A‐MXene to fabricate a superlattice GerMXene heterostructure. As‐synthesized GerMXene exhibits high surface area with concentrated functional groups and negative surface charge, which contributes to its excellent dispersibility and stability in aqueous media, optical absorption and bioactivity. A schematic model depicting the synthesis and atomic structure of multidimensional GerMXene heterostructure is shown in **Figure** **1** and Figure S1 (Supporting Information).

## 2 Results and Discussion

### 2.1 Development of A‐MXene Complex (Ti_3_C_2_O/—OH/—O(OH)/—(OH)_2_/—F/Ti Oxides)

**Figure 2:**
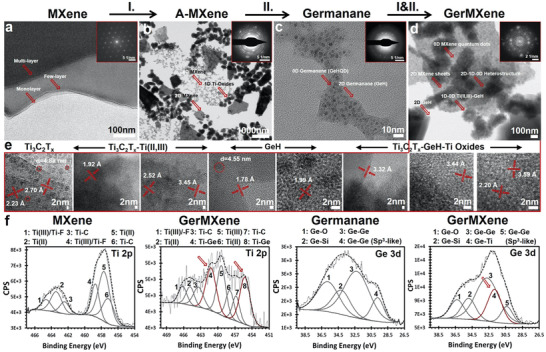
Synthesis and microstructural characterization of multidimensional GerMXene superlattice heterostructure. TEM images and corresponding SAED patterns (insets) of different composites. a) 2D Ti_3_C_2_T*_x_* MXene nanosheets. b) Crystalline A‐MXene complex. c) 2D‐0D hydrogenated GeH nanosheets and derived quantum dots. d) Multidimensional GerMXene superlattice. High‐resolution TEM images displayed the lattice of distinct crystals formed in the structure of GerMXene material. Our data confirmed the successful synthesis of GerMXene with unique morphology and microstructure of 2D MXene in a multidimensional material, including Ti_3_C_2_T*_x_* nanosheets and quantum dots, GeH nanosheets and quantum dots, and surface titanium oxide nanoparticles. e) These data clearly showed the lateral characteristic of different crystals in the structure of GerMXene with d‐spacing ranged from 1.780 to 3.590 Å. f) The XPS narrow scan spectra of Ti 2p and Ge 3d corresponding to Ti_3_C_2_T*_x_* MXene, GeH, and GerMXene. XPS data confirmed an exact reaction between titanium and germanium (Ge=Ti=Ge) to form GerMXene superlattice heterostructure. Further, our XPS analysis clearly showed the emergence of new Ti–Ge peaks in the spectra of Ti 2p and Ge 3d at the binding energy of 452–472 and 27–39 eV, respectively. These findings confirm that GeH quantum dots derived from hydrogenated GeH nanosheets are embedded in the structure of A‐MXene and form stable titanium germanide heterostructure.

The aqueous dispersion of multi, oligo and mono‐layered Ti_3_C_2_T*_x_* MXene nanosheets, followed by steam treatment at 121 °C at a pressure of 134 kPa for 30 min led to formation of A‐MXene crystals. The transmission electron microscopy (TEM) and selected area electron diffraction (SAED) images of the A‐MXene revealed the formation of additional crystal lattice distortions in the microstructure of Ti_3_C_2_T*_x_* MXene nanosheets (**Figure** **2**a,b; Figure S2, Supporting Information). Our synthesis protocol resulted in the direct conversion of 2D Ti_3_C_2_T*_x_* MXene to a unique composite material with enhanced surface properties compared to its pristine nanosheets. Additionally, A‐MXene displayed the microstructural characteristics of Ti_3_C_2_T*_x_* nanosheets, quantum dots and surface titanium oxide nanoparticles in a single material. Our TEM/SAED data of Ti_3_C_2_T*_x_* MXene nanosheets displayed single lattice fringes with d‐spacing of ≈2.70 Å assigned to the hexagonal crystalline planes (‐1100, 01–10, and 10–10) of MXene nanosheets (Figure 2a, inset). These findings are in line with the previous reports on Ti_3_C_2_T*_x_* MXene nanosheets.^[^ ^13^, ^42^ ^]^ The SAED pattern of A‐MXene confirmed that the basal planes’ hexagonal structure of Ti_3_C_2_T*_x_* nanosheets remained unchanged after hydrothermal treatment (Figure 2b, inset). However, the TEM/SAED data of A‐MXene confirmed the emergence of Ti_3_C_2_T*_x_* quantum dots and surface titanium (II) and titanium (III) oxide nanoparticles into MXene nanosheets through secondary nucleation using hydrothermal process (Figure 2b, inset). Additionally, the crystalline pattern of A‐MXene displayed diffusion effect of titanium on the surface of MXene nanocrystals with polycrystalline diffraction rings corresponding to the (101), (103), (200), (211), (208), and (215) planes. The proposed chemical reactions for the formation of A‐MXene are presented in Equations S1–S4 in the Supporting Information. Taken together, the A‐MXene heterostructure that was obtained in the current study demonstrated a unique morphology of Ti_3_C_2_T*_x_* nanosheets which was anchored by different particles of 2D–1D–0D dimensionality.

As described above, the A‐MXene material contains significant amounts of Ti_3_C_2_T*_x_* quantum dots and titanium (II) and titanium (III) oxide nanoparticles, formed and distributed on the MXene surface. The frequency histograms and interquartile range (IQR) analysis of A‐MXene showed that the medians of particles are 4.88 nm in diameter (IQR: 3.675) and 205 nm (IQR: 220), respectively (Figure S3, Supporting Information). Notably, our data showed that this specific morphology of A‐MXene was obtained after hydrothermal treatment and bath sonication of Ti_3_C_2_T*_x_* dispersions. We also characterized the aqueous colloids of exfoliated Ti_3_C_2_T*_x_* sheets before and after hydrothermal treatment and after sonication (Figure 2b; Figures S4 and S5, Supporting Information). Interestingly, our scanning electron microscopy (SEM) and energy‐dispersive X‐ray spectroscopy (EDS) data showed a remarkable difference between microstructure of these samples. In particular, the SEM and EDS images of A‐MXene showed an expansion of Ti_3_C_2_T*_x_* layers with high secondary crystallization and formation of unique particle geometry into MXene nanosheets (Figure S5, Supporting Information). However, spontaneous formation of Ti_3_C_2_T*_x_* quantum dots and surface titanium oxide particles was relatively lower in the sample that was initially treated by sonication. This outcome might be due to the effect of hydrothermal treatment to further intercalate and oxidize MXene nanosheets before mechanical ultrasonic vibration.

Further, we performed X‐ray diffraction (XRD) analysis of Ti_3_C_2_T*_x_* MXene powder and its aqueous colloids. The XRD patterns demonstrated that dispersion of Ti_3_C_2_T*_x_* MXene sheets in aqueous media resulted in relative oxidation and hydrolysis of nanosheets to form stable titanium oxides. We also characterized the crystalline pattern of A‐MXene to determine its phase behavior when dispersed in water. Furthermore, we assessed the phase comparison of A‐MXene with pure Ti_3_C_2_T*_x_* MXene quantum dots. Interestingly, the XRD spectra of A‐MXene confirmed the presence (002) peak at around 2‐theta = 7° as the main characteristic of Ti_3_C_2_T*_x_*. Furthermore, additional peaks were identified in these samples corresponding to the formation of stable phases of titanium oxides on Ti_3_C_2_T*_x_* MXene surface. Notably, the XRD patterns of A‐MXene depicted removal of aluminum from the structure of Ti_3_C_2_T*_x_* MXene nanosheets and quantum dots (Figure S6, Supporting Information).

### 2.2 Development of GeH Quantum Dots

Germanene nanosheets possess relatively weak π‐bonding between germanium atoms, therefore functionalization methods are used to improve its structural and electronic properties. These functionalizations include embedding hydrogen, fluorine and chlorine groups into the structure of germanene. These modifications such as hydrogenation of germanene to GeH increase buckling of the end product. However, dispersibility of GeH flakes in aqueous media to get a uniform and stable colloidal solution is still very challenging to achieve. Because 2D materials with bigger flake size are more prone to wrinkling and precipitation. On the other hand, quantum dots because of their sphericity and small size are easy to disperse and form a colloidal suspension. Therefore, in the current study, we converted 2D GeH sheets to quantum dots with lower dimensionality.

The TEM analysis of hydrogenated GeH nanocrystals revealed a distribution of quantum dots with an average diameter of ≈4.45 nm (SD: 0.770) into its planar structure (Figure S7, Supporting Information). The SAED pattern of GeH was characterized using reciprocal lattice points grown on (1120) and (0002) planes with an angle of 90° (Figure 2c, inset). Besides, we performed the XRD characterization of GeH nanosheets and quantum dots. As shown in Figure S8 in the Supporting Information, the XRD pattern of hydrogenated GeH nanosheets is almost similar to the pattern recorded for aqueous GeH quantum dots. At ≈2‐theta = 16°, a high‐intensity peak of GeH that has been identified as the dominant peak in the XRD spectrum of nanosheets and quantum dots corresponds to (002) planes. These data are in line with the previous reports on GeH material. Additionally, the XRD pattern of GeH exhibited a relatively small peak at ≈2‐theta = 28° corresponding to (100) plane. However, few minor peaks at 2‐theta of ≈27°, 34°, 45°, and 54° of GeH nanosheets are absent in the XRD spectra of quantum dots. These peaks belong to germanium traces that were significantly eliminated from the XRD of GeH quantum dots due to purification during synthesis process. Further physicochemical characterization including SEM, TEM, STEM, EDS, and elemental mapping of 2D GeH material and its derived quantum dots are presented in Figure S9 in the Supporting Information.

### 2.3 Development of Multidimensional GerMXene Superlattice (Ge=Ti=Ge)

The A‐MXene and GeH complexes were incorporated to synthesize a novel multidimensional GerMXene structure by spontaneous reaction of titanium and germanium. This synthesis process efficiently produced a superlattice heterostructure through rapid and spontaneous secondary nucleation. As shown in our TEM and fast Fourier transform (FFT) images, several crystalline lattices were observed in the structure of GerMXene, which were attributed to Ti_3_C_2_T*_x_* MXene nanosheets, quantum dots as well as GeH nanosheets, quantum dots and surface titanium oxide nanoparticles (Figure 2d and inset). The multidimensional GerMXene possesses defined characteristics of its metal carbide, metal oxide and hydrogenated metalloid components with unique raspberry‐like nanoparticles anchored on its surface. Additionally, the FFT pattern of GerMXene material implied multiple circular cases, generally growing in a hexagonal (√3 × √3) superstructure. Furthermore, GerMXene heterostructure benefited from multiple Dirac points of embedded hydrogenated GeH quantum dots in its composition. However, this configuration appears to be less circular in shape and closer to trigonal positions, far away from Dirac points when interacting with the nearest conical bands.^[^ ^43^, ^44^, ^45^ ^]^ Besides, our high‐resolution TEM images demonstrated distinct crystalline lattices in the range of 1.78–3.79 Å, formed during the synthesis of GerMXene (Figure 2e). Further, TEM analyses of multidimensional GerMXene heterostructure are presented in Figure S10 in the Supporting Information.

**Figure 3:**
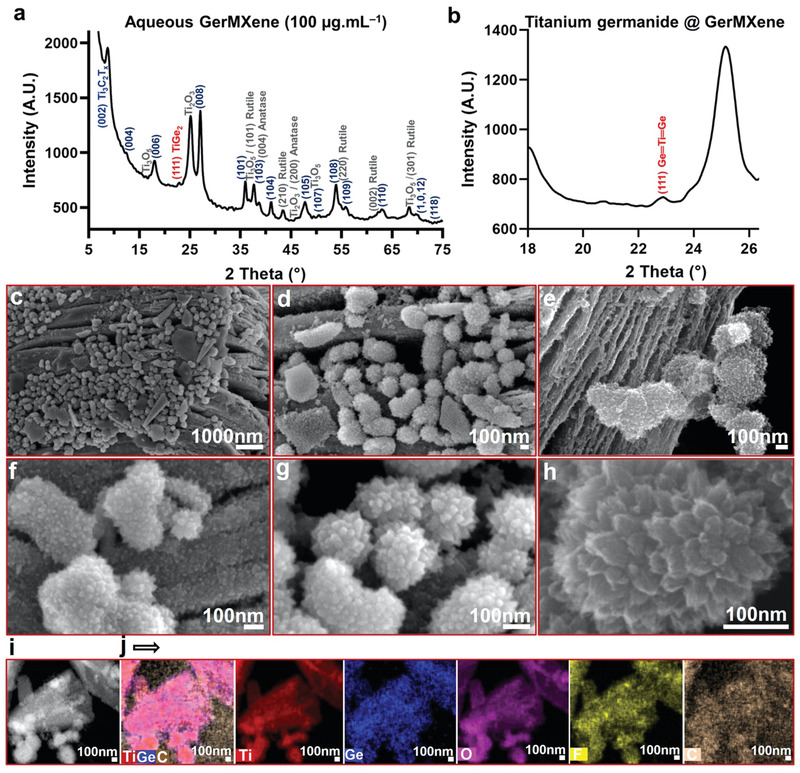
Structural and morphological characterization of GerMXene. a,b) The XRD analysis of multidimensional GerMXene showing structural characteristics of Ti_3_C_2_T*_X_* MXene, hydrogenated GeH and stable titanium oxides. Furthermore, the XRD pattern confirmed the formation of (111) peak at 2‐theta of ≈23° corresponding to titanium germanide bond. The XRD data of GerMXene is in agreement with the XPS analysis of this sample. c–h) SEM images of GerMXene heterostructure at different magnifications. Morphology of the material revealed the conversion of exfoliated 2D Ti_3_C_2_T*_x_* MXene nanosheets to a new multidimensional heterostructure anchored by unique raspberry‐like nanoparticles. In particular, GerMXene superstructure includes significant amounts of MXene nanosheets, MXene quantum dots, germanane nanosheets, germanane quantum dots, and stable titanium oxide nanoparticles in a single material. i,j) Scanning TEM and EDS images of crystalline GerMXene superlattice showing its multidimensional architecture and chemical composition. Our EDS mapping showed the elemental distribution of GerMXene. This analysis confirmed that titanium, germanium, carbon, oxygen, and fluorine are the main components of GerMXene.

Next, the elemental chemical state of GerMXene heterostructure was characterized by X‐ray photoelectron spectroscopy (XPS). In particular, we intended to show the differences in the chemical composition of MXene and GerMXene materials. As described earlier, the Ti_3_C_2_T*_x_* nanosheets were significantly affected by the hydrothermal treatment and covalent bond formation between MXene quantum dots and surface titanium oxide particles. This finding is further confirmed by XPS narrow scan analysis (Figure 2f). The Ti 2p spectrum of Ti_3_C_2_T*_x_* MXene identified the Ti–C, Ti 2p (1/2, 3/2), and Ti (II, III) peaks at binding energies of 457.16–464.61 eV, confirming the successful synthesis of MXene nanosheets. However, the XPS spectra of GerMXene exhibited a significant difference in its surface chemistry compared to MXene sample (Figure 2f). In the Ge 3d narrow scan region of GeH crystals, dominant peaks of Ge–O, Ge–Ge, and sp^3^‐like Ge–Ge were seen at the binding energies of 30.33–36.29 eV. Interestingly, the XPS Ti 2p and Ge 3d analysis of GerMXene showed new titanium germanide peaks at the binding energy of 455.24, 461.21, and 31.80 eV, respectively. This finding was also confirmed by our XRD data of GerMXene. A new peak was detected at 2‐theta around 22° which corresponds to (111) plane of TiGe_2_ bonds (**Figure** **3**a,b). Notably, the other dominant peaks of titanium germanide, including (311), (113), (002) (313), and (600) at 2‐theta of 35–50° were covered by high‐intensity Ti_3_C_2_T*_x_* peaks. These exciting data are in line with our proposed chemical reactions as the first experimental evidence for synthesis of quantum manipulated GerMXene heterostructure. Notably, the peak intensity in the XPS Ge 3d spectra of GerMXene is significantly higher than Ge 3d of GeH material. This phenomenon is a further confirmation of successful fabrication of GerMXene with a high concentration of GeH composition in its atomic structure. The details of XPS peak positions and quantifications are listed in Table S1 in the Supporting Information.

We further characterized the obtained unique surface topography of GerMXene superlattice heterostructure by SEM (Figure 3c–g). The surface functionalized nanosheets were anchored by the heterogeneous distribution of Ti_3_C_2_T*_x_*, GeH and titanium oxide particles. Our SEM data depicted that raspberry‐like nanoparticles grown on the surface of GerMXene have an average diameter of 211.264 nm (SD: 53.912). Additionally, it was observed that the median interlayer distance of nanosheets in the MXene was significantly decreased from 140 nm (IQR: 270) to 64.34 nm (IQR: 325.99) in GerMXene material (Figure S11, Supporting Information). The scanning TEM (STEM) image and corresponding EDS mappings of GerMXene displayed a bright crystalline surface of material and its compositional elements (Figure 3i,j). The morphological details of GerMXene were further characterized by SEM images at different magnifications as presented in Figure S12 in the Supporting Information.

### 2.4 Chemical Reactions and Phase Analysis of Model

Next, we described the possible chemical reaction and phase analysis for the formation of GerMXene. The unique GerMXene heterostructure may have grown due to the spontaneous and intrinsic tendency of titanium and germanium to form titanium germanide (TiGe_2_ and Ti_6_Ge_5_) bonds. These covalent reactions take place at room temperature and the resultant buckled material possesses excellent dispersibility in aqueous solutions. Hence, we described a reaction model between titanium and metalloid germanium that can occur with a small free energy change, which is possible through two chemical procedures. First, typical diffusion‐controlled and nucleation reactions allow the crystallization of Ti_6_Ge_5_ structure at low temperatures. After this reaction, the resultant titanium germanide bonds will interact with the remaining germanium atoms to construct TiGe_2_ bond due to direct primary or secondary nucleation mechanism (Equations S5–S8, Supporting Information). Our proposed reactions for the formation of titanium germanide are effective at room temperature and stable at higher temperatures of up to 650 °C. This is further supported by recent computational simulations based theoretical investigations.^[^ ^46^, ^47^ ^]^ Therefore, the experimental model in the current study and theoretical evidence in literature strongly suggest the occurrence of a possible reaction between group 4 or 5 transition metals and group IVA metalloids.

Next, to enhance the scope of the current study, we performed literature search to explore the possibility of occurrence of similar reactions with other MXenes and metals of similar structures such as tantalum‐germanide (TaGe_2_), niobium‐germanide (NbGe_2_), titanium silicide (TiSi_2_), vanadium silicide (V_6_Si_5_), vanadium tantalum silicide (Ta_2_V_4_Si_5_) and niobium chromium silicide (Nb_4_Cr_4_Si_5_). We found that computational simulations based theoretical studies (Figures S13–S16, Supporting Information) have reported that the dissimilarity values for the formation of these structures are between 0.18 and 0.45 that highlights the potential of our experimental protocols in the current study to synthesize new materials with other MXenes.^[^ ^48^ ^]^ In case of GerMXene, TiGe_2_ crystal structure is in the orthorhombic Fddd space‐group and the spread Ti–Ge or Ge–Ti and Ge–Ge bond distances are in the range of 2.65–2.93 Å and 2.66–2.92 Å, respectively. Additionally, the formation of Ti_6_Ge_5_ crystals appears in the orthorhombic Ibam space‐group with three titanium sites. First, due to the spreading of Ti–Ge or Ge–Ti bonds at 2.63–2.95 Å, each titanium atom binds to seven atoms of germanium to generate a TiGe_7_ pentagonal pyramid structure. Similarly, at the other sites, titanium bonds with the other seven and six germanium atoms. Also, in the first inequivalent site of germanium, it readily bonds with eight titanium atoms. In the second and third sites, the germanium atoms with a length of 2.62 Å bond with other nine and seven titanium atoms, respectively.^[^ ^49^, ^50^, ^51^ ^]^ The calculations for crystal model, formation energy, band structure and density of states along with their phase diagram, average absorption and elasticity are provided in Figures S13–S16 in the Supporting Information. The orthorhombic TiGe_2_ crystal represents Fddd space group (Hermann–Mauguin symbol [70]) with an approximate density of 6.55 g·cm^−3^, formation energy (ΔH_f_) of —0.423 eV, decomposition energy of 0.013 eV, energy‐above Hull of 0.000 eV per atom and the defined lattice parameters of volume is 97.874 Å^3^ (*a* = 6.202, *b* = 5.111, *c* = 5.030).^[^ ^47^, ^52^, ^53^ ^]^ For Ti_6_Ge_5_ structure with a lattice of 354.398 Å^3^, the similar orthorhombic crystal with a space group Ibam (72) and a density of 6.09 g·cm^−3^ could form at formation energy, decomposition energy of 0.042 eV, energy above Hull of –0.666 and 0.011 eV per atom, respectively. Taken together, the data in the current study provide an experimental reaction model to synthesize new MXene‐based heterostructures.

### 2.5 Bioactivity and Biocompatibility of GerMXene

**Figure 4:**
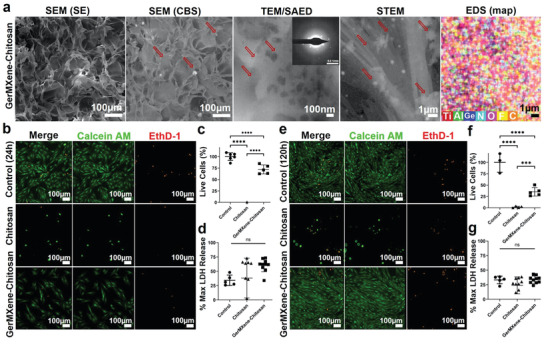
Characterization of GerMXene–chitosan and chitosan scaffolds and assessment of biocompatibility with H9C2 cells. a) The SEM/TEM/SAED images and EDS mapping of the crosslinked GerMXene–chitosan hydrogel was performed. The backscatter images clearly showed a distribution of GerMXene in the polymeric matrix of chitosan. The SAED pattern depicted crystalline rings of GerMXene–chitosan. Furthermore, the backscatter TEM images confirmed a successful synthesis of multidimensional GerMXene–chitosan heterostructure. Also, EDS mapping further displayed an elemental composition of GerMxene–chitosan. b–e) The biocompatibility and bioactivity of b,c) chitosan hydrogel and d,e) GerMXene–chitosan composite hydrogels was assessed after co‐culture with H9C2 cells for 24 and 120 h (*n* = 5 per group). After the co‐culture live/dead assay was performed in H9C2 cells. The cells were stained with green Calcein AM for live cells and red EthD‐1 for detecting dead cells. There was a significant increase in the proliferation of H9C2 cells after co‐culture with GerMXene–chitosan on day 5. Imaging of the cells was carried out using Nikon Ti‐2 fluorescent microscope. The cytotoxicity evaluation of chitosan and GerMXene–chitosan hydrogels was performed after co‐culture with H9C2 cells for 24 h using LDH assay. Our data revealed no significant increase in LDH release after co‐culture with the materials for 24 h (*n* = 5‐8 per group). (“ns” = statistically no significant difference, *** = *p* < 0.001, and **** *p* < 0.0001).

Next, we determined the biocompatibility and potential of GerMXene for tissue engineering and regenerative medicine applications. Recently, there has been growing interest in the application of carbon nanomaterials containing scaffolds to mimic the native extracellular matrix (ECM) to facilitate cellular attachment, proliferation and signaling.^[^ ^54^, ^55^ ^]^ In this regard, we added colloidal suspensions of GerMXene (100 µg·mL^−1^) into clinically approved chitosan hydrogel to synthesize 3D injectable scaffolds. The detailed preparation and physicochemical characterization of chitosan and GerMXene–chitosan hydrogels is described in the method section. We characterized the microstructural properties of pristine and composite scaffolds by SEM, TEM, EDS, and Fourier‐transform infrared (FTIR) spectroscopy. As shown in **Figure** **4**a, the hydrogels are uniformly crosslinked with the median pore size of 51.55µm (IQR: 85.23) in chitosan samples. Notably, an increase in the pore size of composite hydrogels to 80.89 µm was observed after incorporation of GerMXene crystals into the chitosan network. These findings were further confirmed by SEM/TEM images and EDS elemental mapping of GerMXene–chitosan samples (Figure 4a; Figures S17 and S18, Supporting Information). Furthermore, our FTIR data showed the emergence of additional peaks in the structure of GerMXene–chitosan with high amount of surface functional groups compared to chitosan samples. The FT‐IR spectra of GerMXene–chitosan presented the surface functional groups including —OH, COOH, C=O, Ti—O, Ti—C, Ti—F, Ge—H, Ge—H_2_, and —NH bonds at a wavelength range of 400–4000 cm^−1^ (Figure S19, Supporting Information).

To evaluate the biocompatibility and bioactive properties of GerMXene, the chitosan and GerMXene–chitosan hydrogels were co‐cultured with H9C2 cells (cardiomyocyte cell line) for 24 and 120 h. Prior to that, we assessed the biocompatibility of aqueous colloids of A‐MXene, GeH quantum dots and GerMXene with H9C2 cells at a concertation of 100 µg·mL^−1^. Our data showed no significant cytotoxic effects of the tested materials in H9C2 cells after 24 h of co‐culture (Figure S20, Supporting Information). Interestingly we found that after 24 h of co‐culture of A‐MXene, GeH and GerMXene dispersions with H9C2 cells, these materials were able to spontaneously enter into the cells without any uptake enhancing techniques (Figure S21, Supporting Information). These findings highlight the bioactivity and ability of GerMXene to transit from blood into specific tissues during future in vivo applications for nanomedicine‐based therapies.

**Figure 5:**
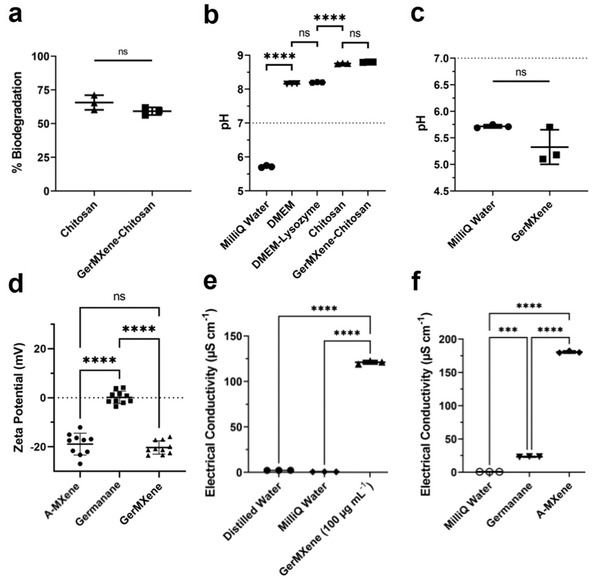
Measurement of biodegradation, pH, surface charge, and electrical conductivity of the synthesized GerMXene hydrogel. a,b) The biodegradability of freeze‐dried GerMXene–chitosan and chitosan hydrogels was measured after 20 days. The results revealed an approximate degradation of 60% in chitosan hydrogels in the presence of lysozyme (*n* = 3). Interestingly, the addition of GerMXene into the hydrogel networks did not affect the biodegradability of chitosan hydrogel. c) pH measurement of GerMXene at a concentration of 1000 µg mL^−1^ (*n* = 3). Our dada displayed no significant changes in the acidity or basicity of MilliQ water in the presence of GerMXene nanocrystals. d) Zeta potential measurement of aqueous GerMXene suspensions at concentration of 100 µg mL^−1^, compared to A‐MXene and germanane samples (*n* = 10). Our data confirmed a negative surface charge behavior of GerMXene at −10 to −25 mV. e,f) The electrical conductivity of aqueous suspension of GerMXene at concentration of 100 µg mL^−1^compared to A‐MXene and GeH quantum dots (*n* = 3).

Furthermore, our data demonstrated that incorporation of GerMXene into the chitosan scaffolds significantly enhanced the cellular attachment and proliferation within the chitosan matrix. These properties are highly desirable for future applications of GerMXene for cell therapy mediated tissue repair. We assessed the survival of H9C2 cells after co‐culture with GerMXene for 120 h, interestingly our data demonstrated a significant increase in the population of H9C2 cells in GerMXene–chitosan hydrogels. There was a remarkable improvement in cell survival and proliferation after 120 h of culture (Figure 4b–g; Figure S22, Supporting Information). Furthermore, the assessment of lactate dehydrogenase (LDH) release confirmed that the material was not cytotoxic to H9C2 cells after 24 h of co‐culture with GerMXene colloids at 100 µg·mL^−1^. Measurement of biodegradation properties of chitosan and GerMXene–chitosan hydrogels demonstrated that the incorporation of GerMXene into hydrogel networks did not affect the biodegradability of chitosan; rather it significantly improved the ability of chitosan to support cell survival and growth (**Figure** **5**a). This functionality is further supported by the pH analysis of freeze‐dried chitosan and GerMXene–chitosan solutions after hydrogels were soaked in culture media for 21 days at 37 °C (Figure 5b). Furthermore, our data showed that the addition of GerMXene did not cause any significant changes in the acidity or basicity of MilliQ water, highlighting its suitability for use in the aqueous applications (Figure 5c). Together, these findings suggest the potential of GerMXene as a bioactive material for tissue engineering and regenerative nanomedicine applications.

### 2.6 Zeta Potential, Electrical Conductivity, and Surface Area of GerMXene

In the next experiment, we measured the surface charge of colloidal suspensions of GerMXene at a concertation of 100 µg·mL^−1^. Our zeta potential (ζ) measurements demonstrated that aqueous GerMXene dispersions have a negative surface charge in the range of −15 to −25 mV (Figure 5d). These data coincide with our measurement on surface charge of synthesized A‐MXene (−10 to −25 mV) and is in agreement with the reports in literature on surface charge properties of 2D MXene nanosheets.^[^ ^56^, ^57^ ^]^ However, our ζ analysis of aqueous GeH colloids showed a significantly lower negative surface charge compared to GerMXene at a similar concentration (+5 to −5 mV). The difference between surface charge of GerMXene and GeH could be due to differences in the surface functional groups. Furthermore, we evaluated the electrical conductivity of aqueous GerMXene (Figure 5e,f). The data showed an average electrical conductivity of ≈125 µS·cm^−1^ of GerMXene samples, which is comparable with A‐MXene (≈175 µS·cm^−1^) and significantly higher than GeH quantum dots (≈25 µS·cm^−1^). Notably, the electrical conductivity of GerMXene remained mostly unchanged after two months.

The surface properties of 2D MXene nanomaterials play an important role in their application in different fields. To characterize the surface properties of GerMXene, we measured specific surface area and porosity by Brunauer‐Emmett‐Teller (BET) nitrogen adsorption‐desorption isotherms and Barrett‐Joyner‐Halenda (BJH) method.^[^ ^23^, ^58^ ^]^ Our BET data demonstrate that GerMXene has a significantly higher surface area compared Ti_3_C_2_T*_x_* MXene nanosheets (Figure S23, Supporting Information). Interestingly, the specific surface area of 2D MXene increased from 7.11 to 91.53 m^2^ g^−1^ in the GerMXene sample. Therefore, conversion of Ti_3_C_2_T*_x_* MXene to GerMXene heterostructure contributed to an approximately 13‐fold increase in the surface area of this material. This structural change is mainly attributed to multidimensionality of GerMXene structure and formation of new particles on the surface of MXene sheets during synthesis. In particular, GerMXene exhibits type‐IV isotherms with H3‐type hysteresis loops characteristic of mesoporous materials in the relative pressure (*P*/P_0_) range of around 0.5–1.0 (Figure S23a, Supporting Information). Notably, our BET analysis is concisely in agreement with the morphological characterization of GerMXene heterostructure (Figure 2d,e, Figure 3c–h; Figure S12, Supporting Information). Furthermore, the total pore volume of GerMXene is also increased from 0.442 cc g^−1^ in MXene to 0.616 cc g^−1^ for GerMXene (Figure S23b, Supporting Information). The employed method, therefore, has effectively produced a new multidimensional superlattice with excellent surface properties.

### 2.7 Optical, Thermal, and Structural Stability of GerMXene

We assessed the optical properties of GerMXene suspensions by ultraviolet‐visible (UV‐Vis) spectroscopy and found well defined lateral carbon structure of Ti_3_C_2_T*_x_* MXene at a wavelength range of 280–900 nm. Furthermore, the UV‐Vis analysis of GerMXene depicted no significant changes in the optical absorption of GerMXene after 60 days of synthesis (Figure S24a, Supporting Information). As shown in the optical micrographs, GerMXene suspensions remained stable without significant agglomeration or stacking at different concentrations (Figures S24b and S25, Supporting Information). The enhanced stability of GerMXene heterostructure can be attributed to the covalent and vdW bonds present in its structure.

The thermal stability of GerMXene was evaluated using thermogravimetric analysis (TGA). After heating for up to 1000 °C under nitrogen atmosphere, GerMXene crystals showed excellent thermophysical stability (Figure S26, Supporting Information). The TGA analysis confirmed no significant changes in the weight percentage of GerMXene material at this temperature with a char residue higher than 97%. However, as expected, at a temperature over 600 °C, a slight decomposition of surface functional groups was observed in the TGA curve of GerMXene powder, resulted in a minor mass‐loss (≈2%).

We further characterized the structural stability of GerMXene in aqueous colloidal suspensions at a concentration of 1000 µg·mL^−1^ by centrifugation at 1500 rpm for 15 min. Our data displayed no significant changes in the morphology and microstructure of GerMXene before and after centrifugation (Figure S27, Supporting Information). We further increased the spinning speed to 3000 rpm for 15 min, our SEM data confirmed that the multidimensional raspberry‐like structure of GerMXene was intact even after high‐speed centrifugation. Furthermore, the stability of GerMXene solids was tested at different temperatures in the range of 4–70 °C. As demonstrated in Figure S28 in the Supporting Information, no significant changes were observed in the structure of GerMXene at tested temperatures. Together, these data support the successful synthesis of a new multidimensional heterostructure nanomaterial with unique morphology and excellent stability for future applications in multiple fields.

## 3 Conclusion

In summary, we have reported a versatile synthesis strategy for converting 2D transition‐metal carbide (M_3_X_2_T*_x_*) MXene sheets to multidimensional GerMXene superlattice heterostructure. The newborn GerMXene possesses a unique microstructure with enhanced surface properties compared to its parent materials, MXene and Xene. Also, GerMXene offers excellent biocompatibility and bioactivity with improved electrical, structural, and optical properties for application in multiple fields.

## 4 Experimental Section

### Synthesis of A‐MXene Complex

The Ti_3_C_2_T*_x_* nanosheets (Laizhou Kai Kai Ceramic Materials Co. Inc.) were dispersed in Milli‐Q water and subjected to autoclave treatment at 121 °C for 30 min. The mixture was allowed to cool at room temperature. The surface modification of multidimensional MXene crystals was further achieved by treating it with ultrasonic treatment at 4 °C for 60–90 min. The obtained material was sterilized and stored at 4 °C for future experiments.

### Synthesis of 0D GeH Quantum Dots

Quantum‐sized dots of GeH were prepared through sonication in an ice bath using 2D hydrogenated GeH (906026, Sigma Aldrich, Canada). Briefly, 0.1 g of GeH flakes were dispersed in 100 mL of isopropyl alcohol (IPA) solution and stirred for one hour for further exfoliation. The mixture was sonicated at 4 °C for 24 h. The dispersion solution was then centrifuged at 3500 rpm for 30 min and thoroughly washed with ultrapure distilled water to obtain GeH crystals in the supernatant. The obtained suspension of GeH nanocrystals was further sonicated at 4 °C for 15–20 days until a desired size of GeH quantum dots was obtained. The colloidal solution was subsequently sterilized and stored at 4 °C for further use.

### Synthesis of Multidimensional GerMXene

The formation of GerMXene heterostructure was achieved through a fast and spontaneous reaction of titanium and germanium at room temperature. Briefly, the aqueous colloidal suspensions of A‐MXene and GeH quantum dots at concentrations of 1000 µg·mL^−1^ (in the ratio of 1:1) were mixed gently. The obtained dispersion was sterilized and stored at room temperature for further characterization and future experiments.

### Synthesis of GerMXene–Chitosan Hydrogels

Chitosan (CS) was used as a base material to prepare hydrogel scaffolds following the in‐house protocols^22^. Briefly, 1 g of UV‐exposed low‐molecular‐weight CS powder (Sigma Aldrich, Canada) was dissolved in 40 mL of 0.1M acetic acid and centrifuged for 10 min at 3000 rpm. Next, the solutions containing β‐glycerophosphate (Calbiochem, 1 g·mL^−1^) and hydroxyethyl cellulose (0.025 g·mL^−1^) were slowly added to the hydrogel under constant stirring at 4 °C. The hydrogel composites were incubated at 37 °C for 30–60 min to obtain viscous polymeric solutions.

### Physicochemical Characterization

The physicochemical properties of materials were characterized by scanning electron microscopy (FEI Nova NanoSEM 450, Thermo Fisher Scientific), transmission electron microscopy (FEI Talos F200X S/TEM, Thermo Fisher Scientific), X‐ray photoelectron spectroscopy (Kratos Axis Ultra XPS) and Fourier‐transform infrared spectroscopy (Thermo Nicolet Nexus 870) at the Manitoba Institute of Materials (MIM), the University of Manitoba. The ultraviolet‐visible spectroscopy and microscopic measurements were carried out by Cytation5 Cell Imaging Multi‐Mode‐Reader (BioTek Instruments) and fluorescence microscope (Nikon Eclipse Ti‐2). The electrical conductivity of material solutions was measured by a DuraProb 4‐Electrode. The pH of the material solution was measured by Thermo Scientific Portable Meter. The surface charge of aqueous GerMXene colloids at a concentration of 100 µg·mL^−1^ was assessed using Nanobrook ZetaPALS (Brookhaven Instruments).

### Surface Area, Zeta Potential, and Thermogravimetric Measurements

The specific surface area of synthesized materials was assessed by Brunauer‐Emmett‐Teller (BET) nitrogen adsorption‐desorption isotherms and Barrett‐Joyner‐Halenda (BJH) method. The surface‐charge of aqueous Ti_3_C_2_T*_x_* MXene nanosheets, germanane quantum dots, and GerMXene colloidal suspensions at a concentration of 100 µg·mL^−1^ was measured using a Brookhaven Nanobrook ZetaPALS Instrument. For thermogravimetric analysis (TGA), a Q‐600 SDT TA‐Instrument was used at a heating rate of 10 °C·min^−1^ in nitrogen (100 mL min). The temperature settings for TGA measurements were as following; it was gradually raised to 100 °C (10 °C/minute), kept constant for 10 min, and then increased to 1000 °C.

### Biodegradability Measurement of Hydrogels

To measure the biodegradability of prepared hydrogels, equal weights (Mi) of freeze‐dried GerMXene–chitosan and chitosan hydrogels were soaked in DMEM containing lysozyme (Sigma Aldrich, CA) at a concentration of 500 µg·mL^−1^ and incubated at 37 °C for 3 weeks. At the end of the incubation period, the CS and composite hydrogels were freeze‐dried to measure the final weight (Mf). The biodegradation rate of the hydrogels was calculated by following equation

(1) $$\begin{array}{c} \text{Biodegradability}\left(\%\right)=\;\frac{M_{i}\;-\;M_{f}}{M_{i}}\times 100 \end{array}$$

### Biocompatibility Assessment

To evaluate the biocompatibility of prepared materials live/dead assay was conducted in H9C2 cells. Briefly, the H9C2 cells were plated in 96 well plates, the synthesized materials were subsequently added to the cultured cells and incubated for 24 and 120 h. The assessment of cell viability was performed using a LIVE/DEAD Viability Kit (L3224, Thermo Fisher Scientific, USA). The quantification was performed using Cytation 5 Cell Imaging Multi‐Mode Reader (BioTek Instruments, USA). The microscopic images were captured using Nikon Eclipse Ti‐2 Fluorescence Microscope (Nikon Instruments Inc., USA). To assess cytotoxicity of synthesized materials toward H9C2 cells, the cells were cultured with different forms of materials at a concentration of 100 µg·mL^−1^ for 24 h. The lactate dehydrogenase (LDH) release from the damaged cells was measured in supernatants using a commercial kit (MK401, Takara Bio).

### Cellular Uptake Assessment of GerMXene

To understand the interaction of GerMXene with H9C2 cells, aqueous suspensions of materials at a concentration of 100 µg·mL^−1^ were co‐cultured with H9C2 cells for 24 h. After the co‐culture the cells were fixed with paraformaldehyde (4%) and mounted using Diamond Antifade reagent (Prolong) containing DAPI (Thermo Fisher Scientific, USA). The images were then captured by a fluorescence microscope (Nikon Eclipse Ti‐2).

### Statistical Analysis

All data in the study were reported as mean ± standard deviation. The statistical comparison between multiple groups was performed using one‐way analysis of ANOVA, followed by Tukey's post‐hoc multiple comparison test and Student's t‐test. In all biological data statistical significance is determined as *p* < 0.05.

## Conflict of Interest

The authors declare no conflict of interest.

## Author Contributions

The study was conceptualized and designed by A.R., A.A., and S.D. A.R., A.A., and W.Y. carried out experiments and acquired data. A.R., A.A., W.Y., H.E., and S.D. interpreted the data and performed statistical and formal analysis. A.R., A.A., W.Y., and S.D. designed the figures. A.R., A.A., and S.D. drafted the manuscript. All authors have read and approved the final manuscript.

## Supporting information

Supporting Information

## Data Availability

The data that support the findings of the current study are available from the corresponding author on reasonable request.
