# Supplementary material for: Conversion of 2D MXene to Multi‐Low‐Dimensional GerMXene Superlattice Heterostructure
Source: Adv Funct Mater. 2021 Nov 30;32(10):2108495. doi: 10.1002/adfm.202108495 (PMC8889893; doi:10.1002/adfm.202108495)
Supplement: Supplementary file 1 — Supporting Information [file ADFM-32-0-s001.pdf]

ADVANCED FUNCTIONAL MATERIALS

Supporting Information

for *Adv. Funct. Mater.,* DOI: 10.1002/adfm.202108495

Conversion of 2D MXene to Multi-Low-Dimensional GerMXene Superlattice Heterostructure

*Alireza Rafieerad, Ahmad Amiri, Weiang Yan, Hossein Eshghi, and Sanjiv Dhingra**

Supplementary Information

Conversion of 2D MXene to multi-low-dimensional GerMXene superlattice heterostructure

Alireza Rafieerad[a], Ahmad Amiri[b], Weiang Yan[a], Hossein Eshghi[c], Sanjiv Dhingra[a*]

[a] *Regenerative Medicine Program, Department of Physiology and Pathophysiology, Rady Faculty of Health Sciences, Institute of Cardiovascular Sciences, St. Boniface Hospital Albrechtsen Research Centre, University of Manitoba, Winnipeg, Manitoba, R2H2A6, Canada*

[b] *J. Mike Walker '66 Mechanical Engineering Department, Texas A&M University, College Station, Texas 77843, United States*

[c] *Department of Chemistry, Faculty of Science, Ferdowsi University of Mashhad, Mashhad, 91775-1436, Iran*

Correspondence:
Sanjiv Dhingra, PhD, FAHA, FAPS
Associate Professor
Regenerative Medicine Program
Director: Canada Italy Tissue Engineering Program
Institute of Cardiovascular Sciences, St. Boniface Hospital Research Centre
R-3028-2, 351 Tache Avenue, Winnipeg, R2H2A6, Canada
Email: sdhingra@sbrc.ca

CONTENTS:

Supplementary Figure S1: Schematic illustration of conversion of transition metal carbide MXene to multi-dimensional GerMXene superlattice heterostructure.

Supplementary Figure S2: Morphology of 2D $Ti_3C_2T_x$ MXene nanosheets and crystalline A-MXene complex.

Supplementary Figure S3: Diameter range of grown particles in the structure of A-MXene.

Supplementary Equations 1-4: Chemical reactions depicting phase-transformation of 2D $Ti_3C_2T_x$ MXene nanosheets to A-MXene complex.

Supplementary Figure S4: Microstructure and elemental characterization of $Ti_3C_2T_x$ MXene and A-MXene complex.

Supplementary Figure S5: Morphology and microstructure of aqueous A-MXene complex and $Ti_3C_2T_x$ MXene dispersions.

Supplementary Figure S6: : XRD patterns of $Ti_3C_2T_x$ MXene powder, aqueous $Ti_3C_2T_x$ sheets and quantum dots as well as A-MXene.

Supplementary Figure S7: Size distribution of synthesized monoelemental hydrogen-terminated germanium quantum dots.

Supplementary Figure S8: XRD crystalline pattern of exfoliated 2D germanane (GeH) material and its derived quantum dots.

Supplementary Figure S9: Morphology and microstructural characterization of 2D hydrogenated GeH nanosheets and 0D GeH quantum dots.

Supplementary Figure S10: Morphological characterization of multi-low-dimensional GerMXene heterostructure.

Supplementary Table S1: Detailed XPS comparison of elemental and peak information of the synthesized materials.

Supplementary Figure S11: Wall-to-wall interlayer measurement distribution of GerMXene superlattices heterostructure.

Supplementary Figure S12: Further morphological characterization of GerMXene heterostructure.

Supplementary Equations 5-8: Description of chemical reaction of titanium and germanium during formation of GerMXene.

Supplementary Figure S13: Database of the model and crystal structure and decomposition energy analysis of titanium germanide bonds.

Supplementary Figure S14: Phase stability prediction, estimated prototype and material properties computation of titanium germanide ($TiGe_2$ and Ti_6Ge_5) bonds.

Supplementary Figure S15: Predicted crystallization and lattice parameters of $TiGe_2$ in the structure of GerMXene superlattice heterostructure.

Supplementary Figure S16: Crystallization model, computed lattice parameters, band structure, the density of states, X-ray diffraction and similar chemistry of Ti_6Ge_5.

Supplementary Figure S17: Structural characterization and chemical composition of the crosslinked GerMXene-chitosan hydrogel networks.

Supplementary Figure S18: Morphology and microstructural characterization of crosslinked chitosan hydrogels.

Supplementary Figure S19: Identification of chemical bonds of MXene and hydrogenated germanane (GeH) nanocrystals as well as GerMXene-chitosan and chitosan hydrogels.

Supplementary Figure S20: Assessment of biocompatibility of aqueous colloidal GerMXene compared to A-MXene and 0D GeH quantum dots suspensions.

Supplementary Figure S21: Assessment of cellular uptake of aqueous GerMXene, GeH quantum dots, and A-MXene collides.

Supplementary Figure S22: Assessment of biocompatibility and bioactivity properties of the GerMXene-chitosan compared to A-MXene-chitosan hydrogel scaffolds.

Supplementary Figure S23: Specific surface area measurements of GerMXene using Brunauer-Emmett-Teller (BET) nitrogen adsorption-desorption isotherms and Barrett-Joyner-Halenda (BJH) method.

Supplementary Figure S24: UV-Vis and optical analysis of aqueous GerMXene colloids at day 1 and day 60 of synthesis.

Supplementary Figure S25: Optical image of aqueous GerMXene colloids at different concentration at room temperature.

Supplementary Figure S26: Thermophysical properties and decomposition resistance of GerMXene superlattice heterostructure.

Supplementary Figure S27: Morphology illustration of precipitated GerMXene colloids after spinning at 1500 rpm for 15 minutes.

Supplementary Figure S28: Morphology characterization of GerMXene material after exposure to different temperatures from 4 °C to 7 °C.

Supplementary Information References: Additional context references.

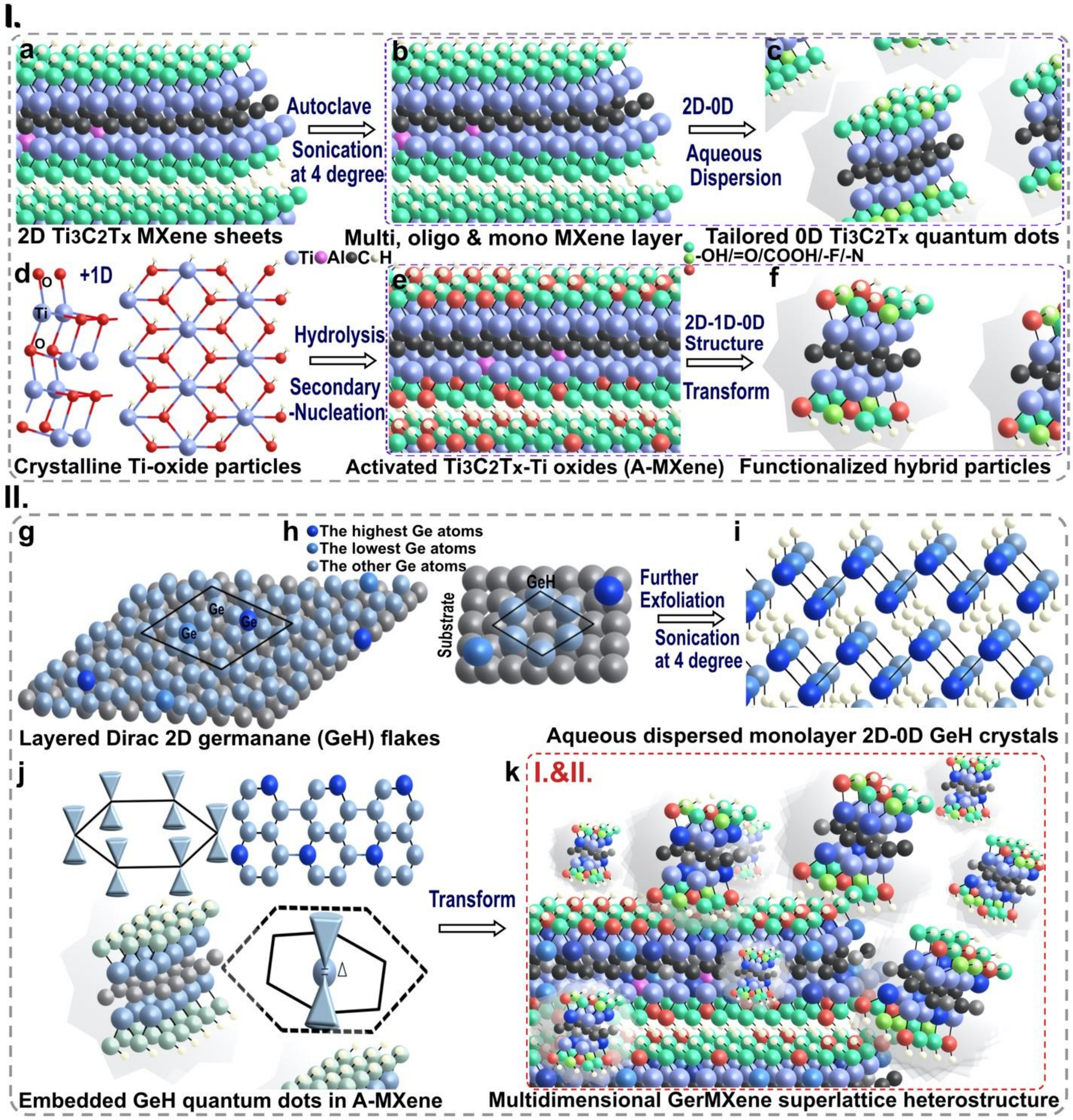

Supplementary Figure S1: Schematic illustration of conversion of transition metal carbide MXene to multi-dimensional GerMXene superlattice heterostructure. a-f, The model depicts treatment of MXene to produce A-MXene complex. Purple, pink, black and beige colors represent titanium, aluminium, carbon and hydrogen respectively. Red and green colored balls represent the functional groups on A-MXene surface. **g-j**, Fabrication of monoelemental 0D GeH quantum dots from 2D hydrogenated GeH nanosheets (dark, light and ultralight blue marked the highest, lowest and other germanium atoms on GeH surface. **k**, van der Waals-covalent assembly of 2D-1D-0D GerMXene heterostructure anchored by raspberry-like nanoparticles at room temperature.

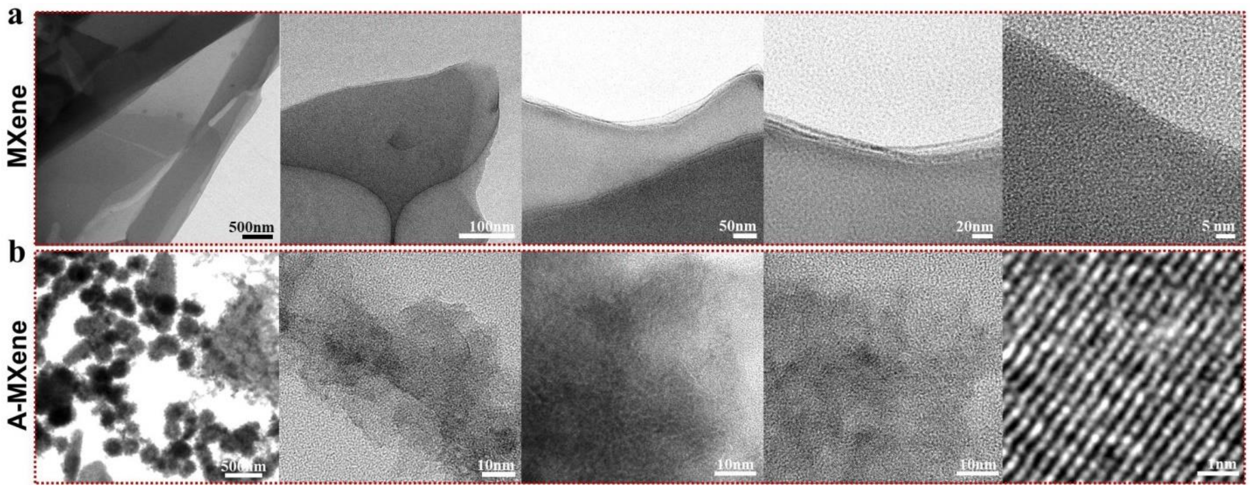

Supplementary Figure S2: Morphology of 2D $Ti_3C_2T_x$ MXene nanosheets and crystalline A-MXene complex. a, TEM images of accordion-like MXene nanosheets at different magnifications. **b**, TEM of A-MXene material, after autoclaving the material at 121 ºC for 30 minutes and bath sonication for 45 minutes. The obtained crystalline A-MXene composites are shown to possess higher stability in solid and aqueous media compared to pristine $T_3C_2T_x$ nanosheets.

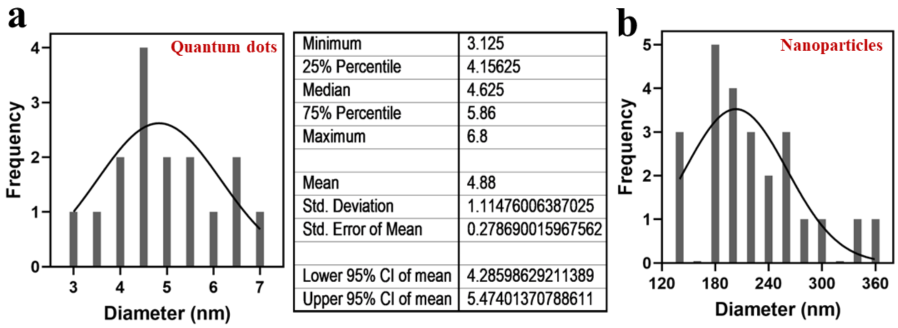

Minimum	3.125
25% Percentile	4.15625
Median	4.625
75% Percentile	5.86
Maximum	6.8
Mean	4.88
Std. Deviation	1.11476006387025
Std. Error of Mean	0.278690015967562
Lower 95% CI of mean	4.28598629211389
Upper 95% CI of mean	5.47401370788611

Minimum	130
25% Percentile	172.5
Median	205
75% Percentile	250
Maximum	350
Mean	216.25
Std. Deviation	59.183759523237
Std. Error of Mean	12.0808343242929
Lower 95% CI of mean	191.25889013484
Upper 95% CI of mean	241.24110986516

Supplementary Figure S3: Diameter range of grown particles in the structure of A-MXene. a-b, The size distribution confirms the presence of two different types of anchored particles into $Ti_3C_2T_x$ nanocrystals. **a**, $Ti_3C_2T_x$ quantum dots and **b**, titanium oxide nanoparticles in the surface.

Supplementary Equations 1-4: Chemical reactions depicting phase-transformation of 2D $Ti_3C_2T_x$ MXene nanosheets to A-MXene complex. Colloidal suspensions of titanium carbide MXene nanosheets were activated by hydrothermal treatment to form A-MXene crystals. The chemical reactions present the formation of a unique morphology of MXene materials that includes $Ti_3C_2T_x$ nanosheets, quantum dots and stable surface titanium oxide nanoparticles as described in the following equations. These reactions are in agreement with the previous reports on the structural change and phase transformation of 2D MXene in aqueous media[1].

(1) $2Ti_3C_2O_2 + 11H_2O \rightarrow 6TiO_2 + CO + CO_2 + 2CH_4 + 7H_2$ Equation 1

(2) $2Ti_3C_2(OH)_2 + 11H_2O \rightarrow 6TiO_2 + CO + CO_2 + 2CH_4 + 9H_2$ Equation 2

(3) $3TiO_2 + 6H_2 \rightarrow 3Ti + 6H_2O$ Equation 3

(4) $2Ti_3C_2O_2 + 5H_2O \rightarrow 3Ti + 3TiO_2 + CO + CO_2 + 2CH_4 + H_2$ Equation 4

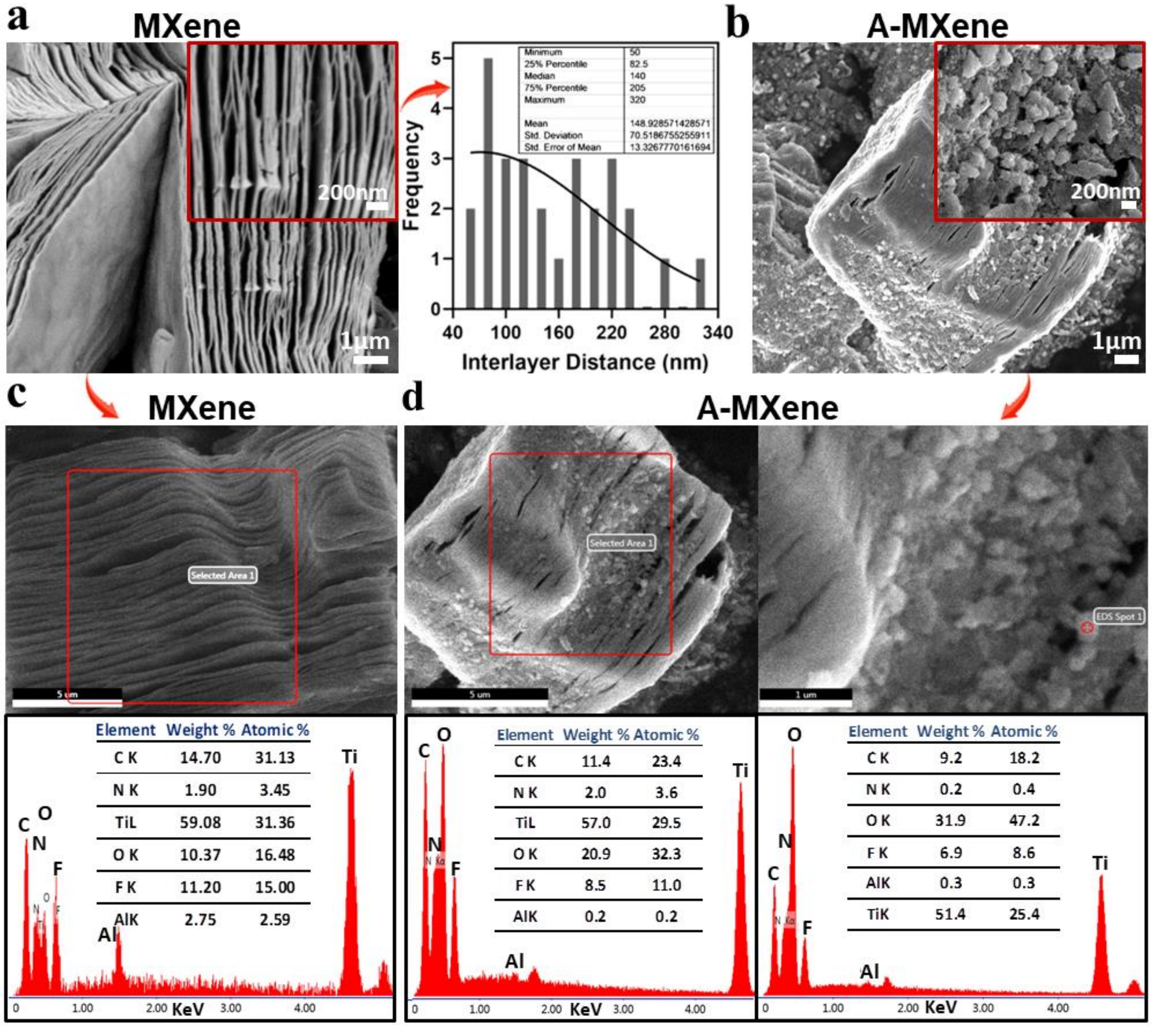

Supplementary Figure S4: Microstructure and elemental characterization of $Ti_3C_2T_x$ MXene and A-MXene complex. a,b, SEM pictures of the prepared materials at different magnifications. **c,d**, EDS/SEM analysis confirmed a significant change in the morphology, microstructure and chemical composition of MXene layers before and after hydrothermal treatment.

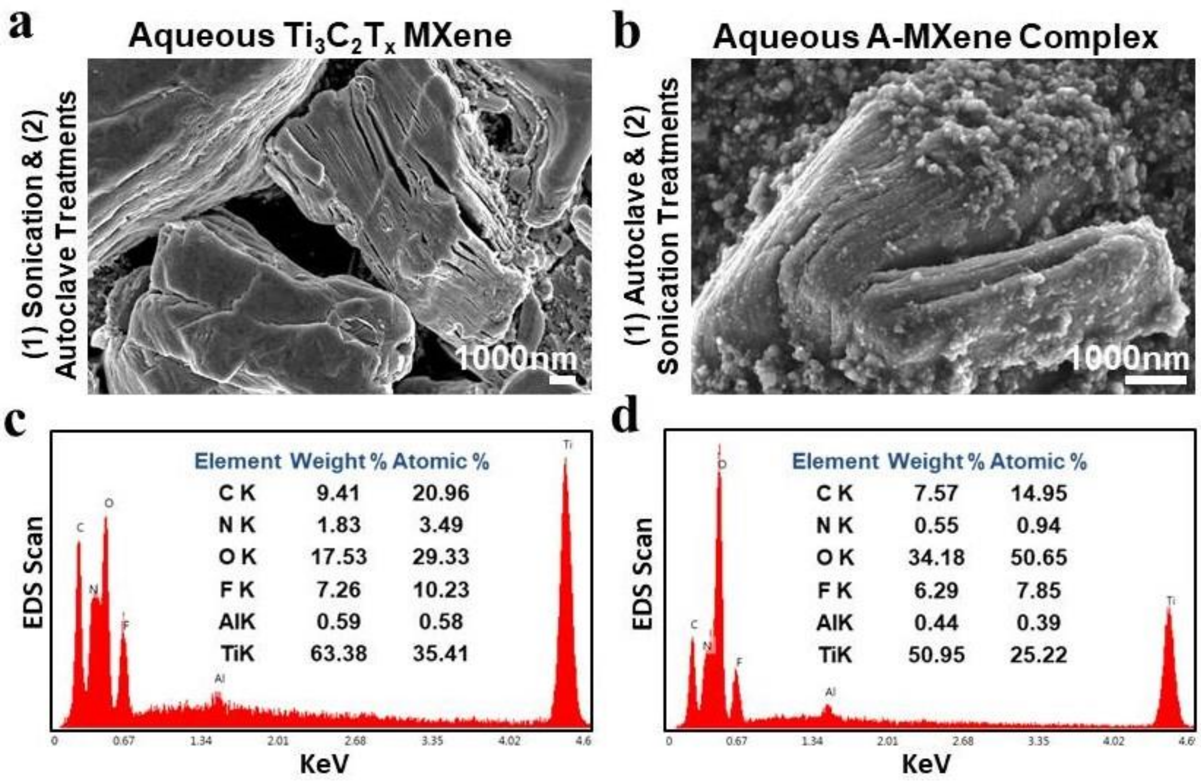

Supplementary Figure S5: Morphology and microstructure of aqueous A-MXene complex and $Ti_3C_2T_x$ MXene. a, The SEM analysis of the synthesized aqueous $Ti_3C_2T_x$ MXene dispersion after sonication. **b**, Characterization of A-MXene samples after autoclave treatment at 121 ºC for 30 minutes and sonication bath for 45minutes confirmed a significantly different crystalline structure of MXene nanosheets. **c-d,** The EDS analysis of $Ti_3C_2T_x$ MXene **(c)** and A-MXene **(d)** dispersions showing elemental composition of materials. There was a significant increase in the atomic and weight percentage of oxygen in A-MXene due to higher functionalization of MXene nanosheets because of formation of $Ti_3C_2T_x$ quantum dots and surface titanium oxides.

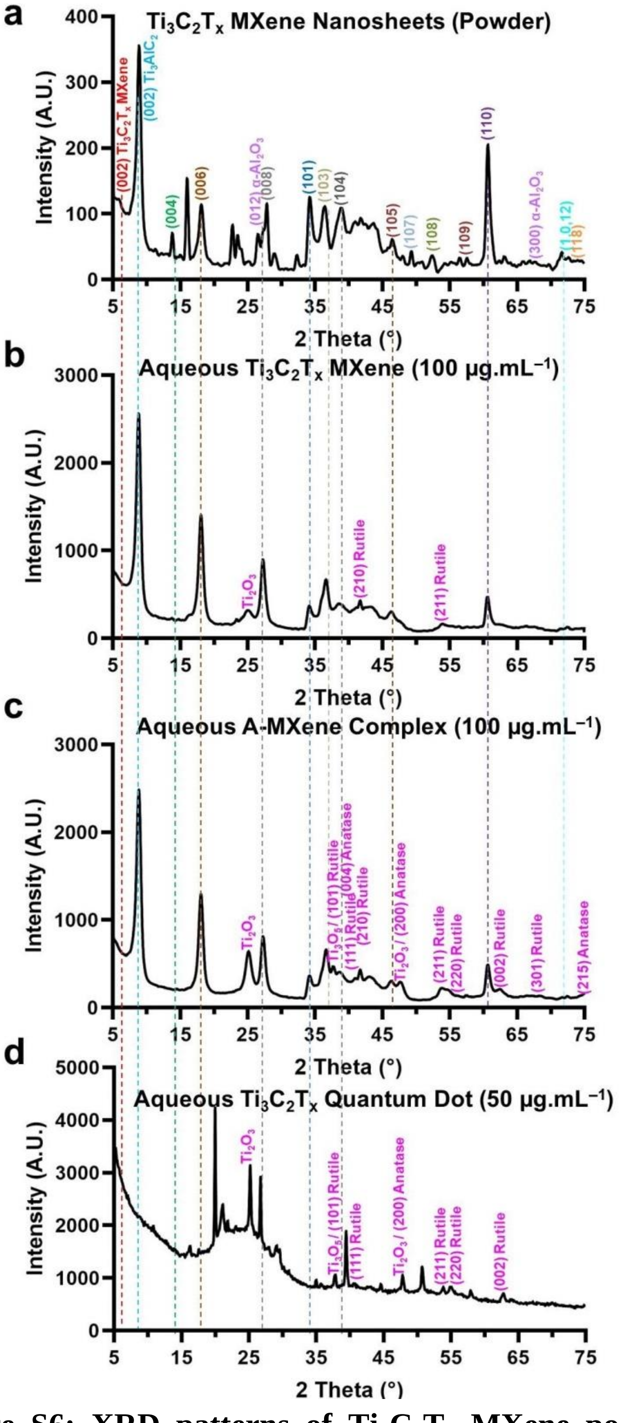

Supplementary Figure S6: XRD patterns of $Ti_3C_2T_x$ MXene powder, aqueous $Ti_3C_2T_x$ sheets, A-MXene and MXene quantum dots. a, XRD analysis of $Ti_3C_2T_x$ MXene confirmed the characteristics of MXene nanosheets with significant extraction of aluminum layers. The XRD spectrum of this sample displayed (002) peaks at 2-theta ~7º. **b**, XRD data of aqueous $Ti_3C_2T_x$ confirmed partial formation of surface titanium oxides on the surface of MXene. **c**, The XRD analysis of A-MXene demonstrated a significant formation of titanium oxide particles on the surface of $Ti_3C_2T_x$ after hydrothermal treatment. **d**, XRD spectra of aqueous $Ti_3C_2T_x$ MXene quantum dots showed higher level of stable surface titanium oxides during synthesis.

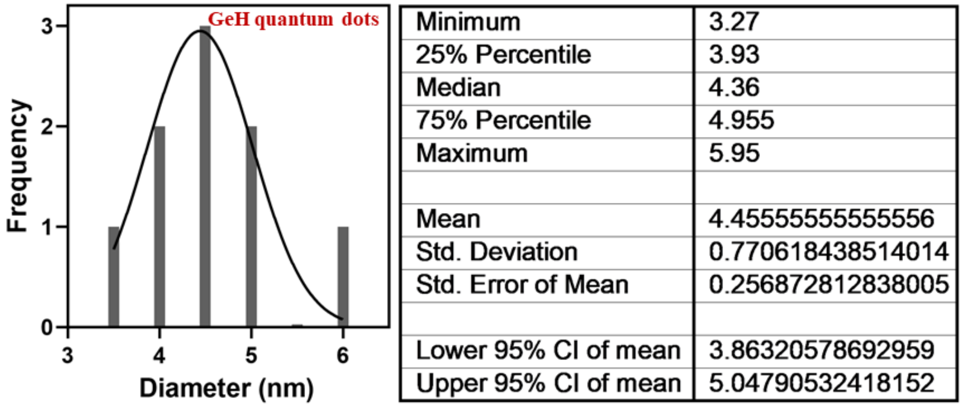

Minimum	3.27
25% Percentile	3.93
Median	4.36
75% Percentile	4.955
Maximum	5.95
Mean	4.45555555555556
Std. Deviation	0.770618438514014
Std. Error of Mean	0.256872812838005
Lower 95% CI of mean	3.86320578692959
Upper 95% CI of mean	5.04790532418152

Supplementary Figure S7: Size distribution of synthesized monoelemental hydrogen-terminated germanium quantum dots. The frequency of data confirmed the quantum-size distribution of 0D GeH particles.

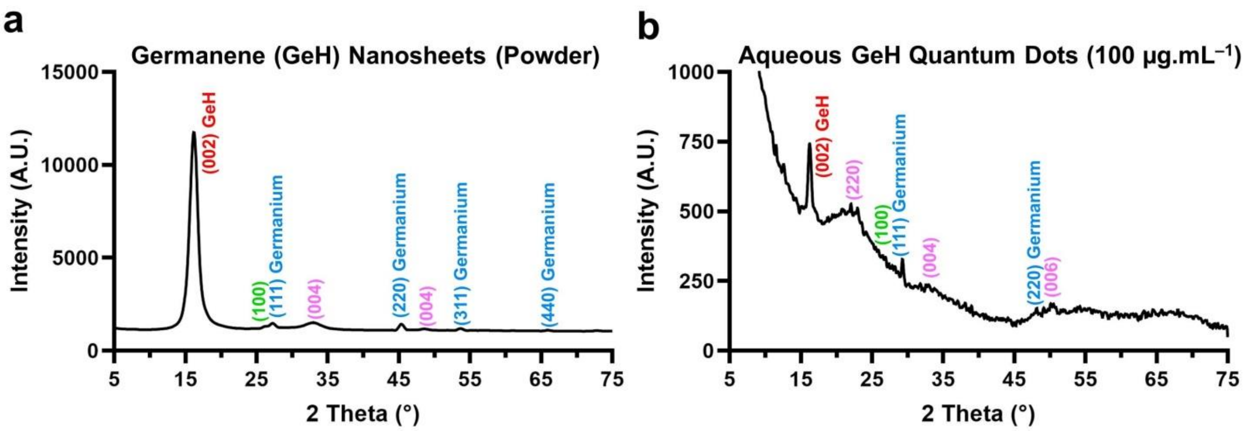

Supplementary Figure S8: XRD patterns of exfoliated 2D germanane (GeH) material and its derived quantum dots. a, The XRD analysis of hydrogenated 2D GeH sheets showing dominant (002) and (110) peaks at 2-theta ~ 16° and 26° respectively. Further, the XRD pattern also includes germanium peaks. **b**, The XRD spectrum of aqueous GeH quantum dots also displayed the original peaks of GeH nanosheets. Our data confirmed the purity and uniformity of GeH quantum dots in aqueous suspensions.

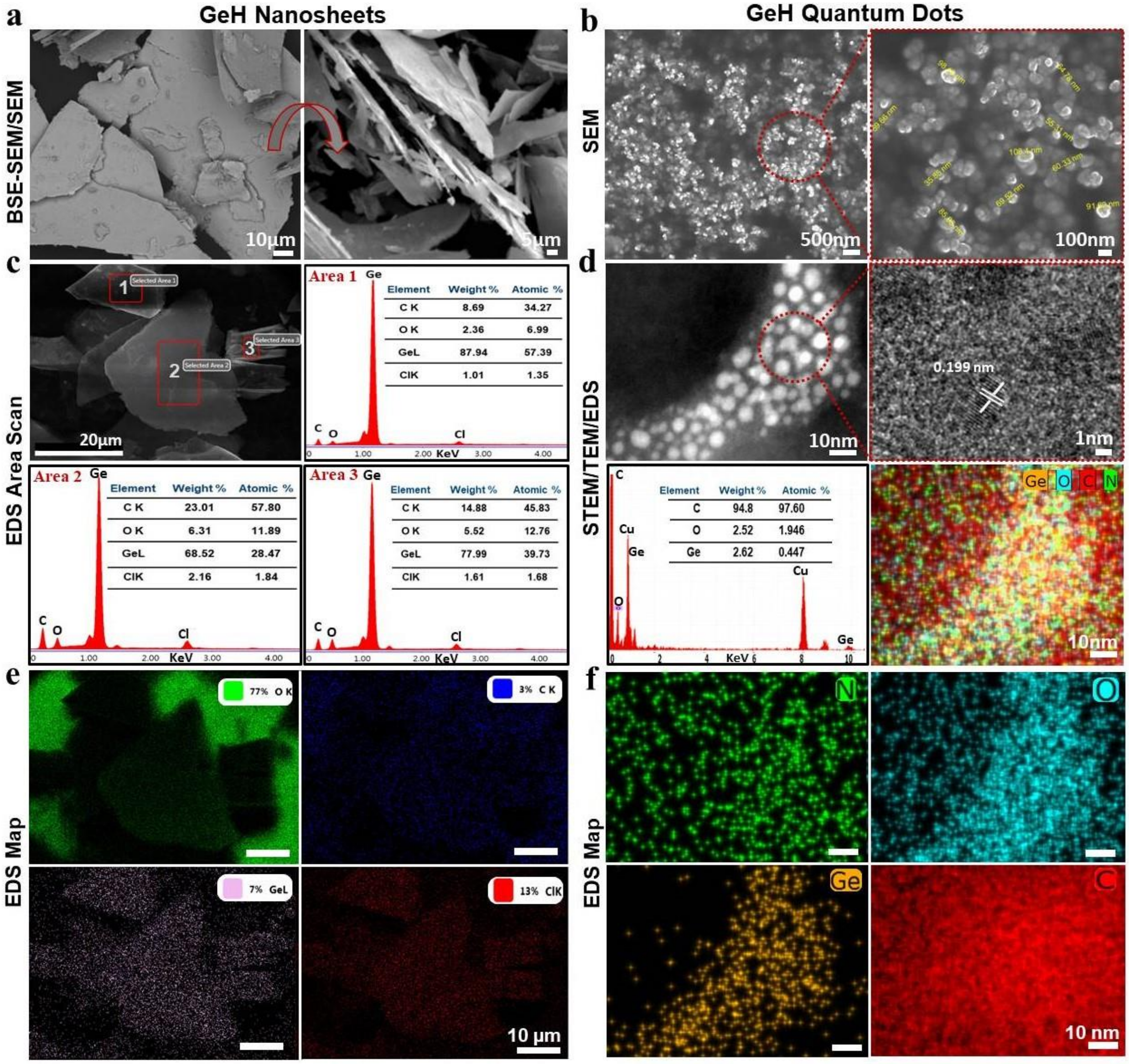

Supplementary Figure S9: Morphology and microstructural characterization of 2D hydrogenated GeH nanosheets and 0D GeH quantum dots. a,b, SEM micrographs of GeH nanosheets and GeH quantum dots. **c**, EDS spectra and monoelemental analysis of GeH nanosheets. **d**, Scanning TEM and HRTEM images of crystalline GeH quantum dots reveled the d-spacing lattice of 0.199 nm. EDS spectra showed the elemental composition analysis of the synthesized GeH particles. **e,f**, EDS elemental mapping of planar GeH and derived quantum dots.

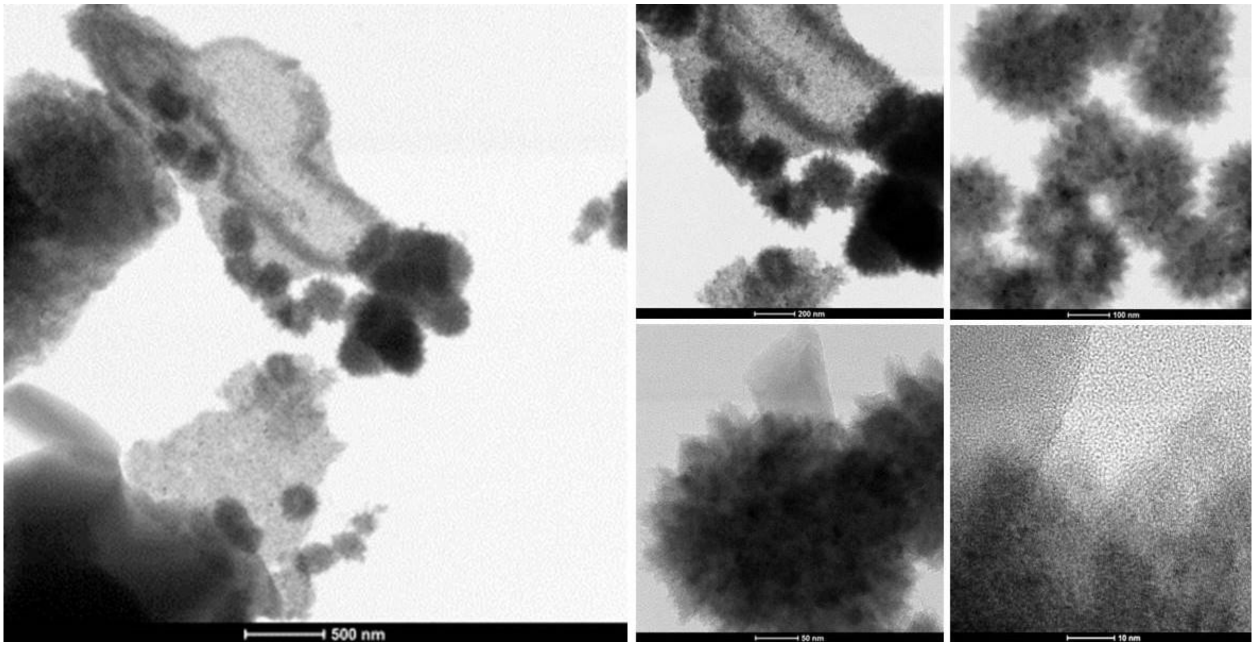

Supplementary Figure S10: Morphological characterization of multi-low-dimensional GerMXene heterostructure. TEM/HRTEM micrographs demonstrating crystalline morphology of titanium carbide, surface titanium oxides as well as germanane nanosheets and quantum dots embedded in a 2D-1D-0D structure.

Supplementary Table S1: XPS analysis to compare the elemental composition and peak information of different materials. The surface analysis of multi-dimensional GerMXene heterostructure showed new peaks, compared to $Ti_3C_2T_x$ MXene and germanane samples.

**Atomic Percentage of Elements**								
**Materials**	**C**	**O**	**Ge**	**Ti**	**Al**	**Si**		
MXene	52.1	40.8	0	6.6	0	0		
Germanane	30.3	29.1	36.3	0	0	3.1		
GerMXene	52	28.1	14	4.1	0	0.8		
**XPS Peaks Information**								
**Ti 2p@ $Ti_3C_2T_x$ MXene**	**Ti-C**	**Ti (II)**	**Ti (III)**	**Ti 2p**	**Ti 2p**	**Ti 2p**		
R.S.F	7.9	7.9	7.9	7.9	7.9	7.9		
Position	457.1606	457.6697	458.7131	462.4213	463.4172	464.6172		
Concentration %	18.35	36.53	16.24	7.29	14.76	6.82		
**Ti 2p@ GerMXene**	**Ti-C**	**Ti (II)**	**Ti (III)**	**Ti-Ge**	**Ti-Ge**	**Ti-C**	**Ti (II)**	**Ti (III)**
R.S.F	7.9	7.9	7.9	7.9	7.9	7.9	7.9	7.9
Position	456.8076	457.8941	459.3817	455.2428	461.2131	463.4072	464.877	466.177
Concentration %	7.97	10.21	19.08	21.23	19.37	8.96	6.74	6.44
**Ge 3d@ Germanane**	**Ge-Ge (sp^3-like)**	**Ge-Ge**	**Ge-Si**	**Ge-O**				
R.S.F	1.55	1.55	1.55	1.55				
Position	29.7412	32.269	34.1368	35.7665				
% Concentration	11.38	45.86	17.13	25.62				
**Ge 3d@ GerMXene**	**Ge-Ge (sp^3-like)**	**Ge-Ge**	**Ge-Si**	**Ge-O**	**Ge-Ti**			
R.S.F	1.55	1.55	1.55	1.55	1.55			
Position	30.3377	32.6991	34.9018	36.29	31.8			
Concentration %	8.84	45.25	13.31	11.92	20.68			

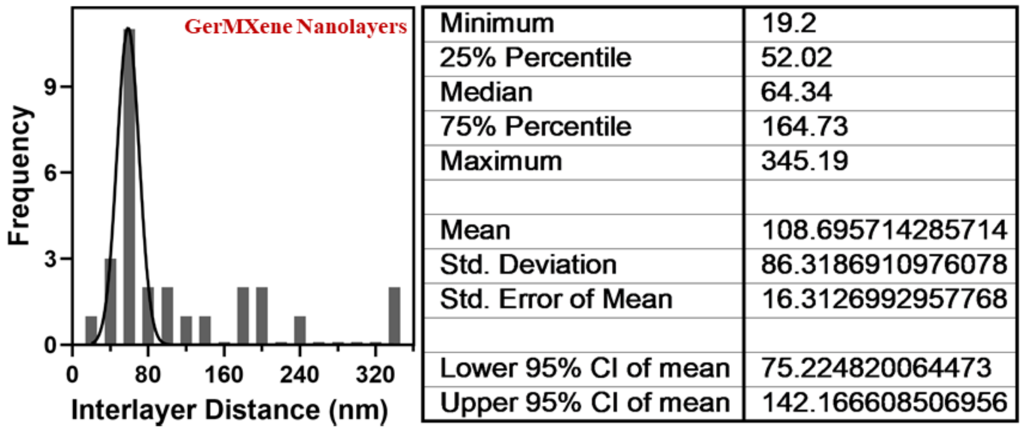

Minimum	19.2
25% Percentile	52.02
Median	64.34
75% Percentile	164.73
Maximum	345.19
Mean	108.695714285714
Std. Deviation	86.3186910976078
Std. Error of Mean	16.3126992957768
Lower 95% CI of mean	75.224820064473
Upper 95% CI of mean	142.166608506956

Supplementary Figure S11: Wall-to-wall interlayer measurement distribution of GerMXene superlattice heterostructure. The frequency of data showed that the interlayer distance of GerMXene was reduced compared to MXene. This phenomenon could be due to the higher secondary nucleation of GerMXene material and embedded quantum dots in its structure.

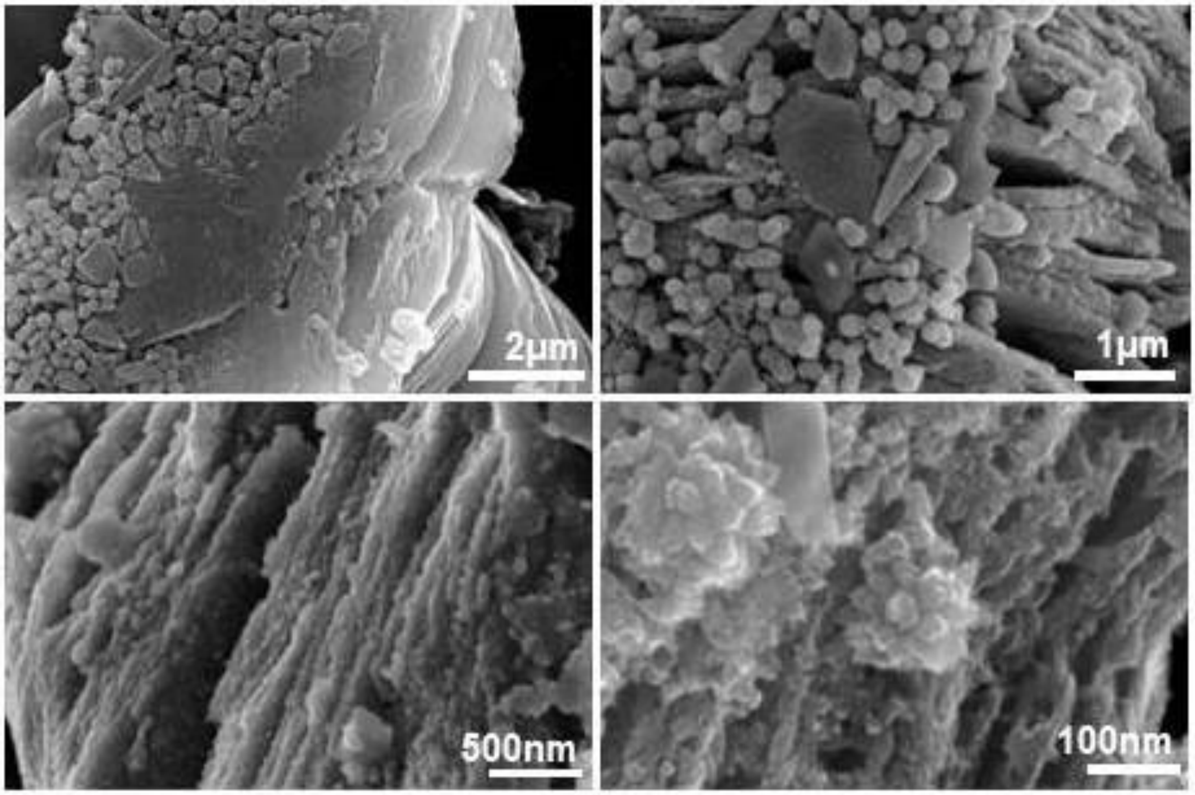

Supplementary Figure S12: Characterization of GerMXene heterostructure. Different magnification SEM images demonstrating the morphological details of the synthesized multi-dimensional GerMXene heterostructure.

Supplementary Equations 5-8: Description of chemical reaction of titanium and germanium during formation of GerMXene. The titanium germanide bonds in the structure of GerMXene were obtained through a rapid and spontaneous reaction between titanium and germanium. This outcome is in agreement with the previous reports on the chemical reaction of these two elements in the literature.[2-5]

(5) $2GeH \rightarrow 2Ge + H_2$ Equation 5

(6) $6Ti + 5Ge \rightarrow Ti_6Ge_5$ Equation 6

(7) $7Ge + Ti_6Ge_5 \rightarrow 6TiGe_2$ Equation 7

(8) $12Ge + 6Ti + Ti_6Ge_5 \rightarrow Ti_6Ge_5 + 6TiGe_2$ Equation 8

$TiGe_2$

Crystal structure

ΔH_f = -0.438 eV/atom

Database Information

Label: icsd-638053

Prototype: TiSi2

Structure: 52475

Spacegroup: Fddd

\# of atoms: 6

Element	x	y	z
Ge	0.581	0.081	0.163
Ge	0.669	0.669	0.337
Ge	0.331	0.331	0.663
Ge	0.919	0.419	0.837
Ti	0	0	0
Ti	0.250	0.750	0.500

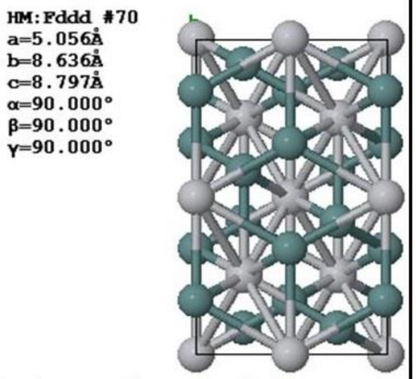

Configuration	Total energy [eV/atom]	Band gap [eV]	Volume [$Å^3$/atom]	ionic steps	Converged
Static	-6.087	0	16.005	1	True
Standard	-6.086	0	15.827	1	True
Fine Relax	-6.075	0	15.827	4	True

ΔH_f = 0.127 eV/atom

Database Information

Prototype: C4_Rutile_TiO2

Structure: 4153849

Spacegroup: P42/mnm

\# of atoms: 6

Element	x	y	z
Ge	0.268	0.268	0
Ge	0.732	0.732	0
Ge	0.768	0.232	0.500
Ge	0.232	0.768	0.500
Ti	0	0	0
Ti	0.500	0.500	0.500

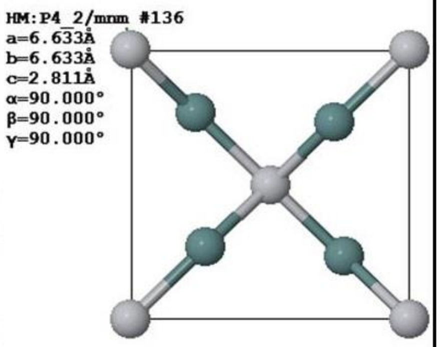

ΔH_f = 1.230 eV/atom

Database Information

Prototype: C9_SiO2

Structure: 4480933

Spacegroup: Fd-3m

\# of atoms: 6

Element	x	y	z
Ge	0.875	0.375	0.375
Ge	0.375	0.875	0.375
Ge	0.375	0.375	0.375
Ge	0.375	0.375	0.875
Ti	0.250	0.250	0.250
Ti	0.500	0.500	0.500

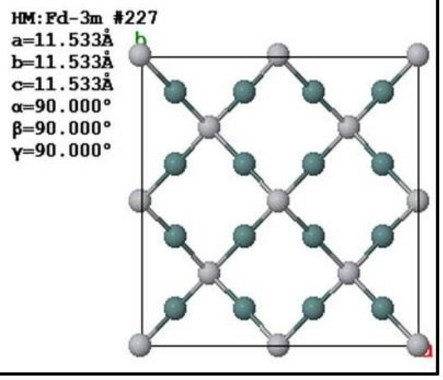

Supplementary Figure S13: Database of the model and crystal structure and decomposition energy analysis of titanium germanide bonds.[6-23] $TiGe_2$ is a three-dimensional titanium disilicide structure with crystallization in the orthorhombic space group of Fddd. In the structure of $TiGe_2$, titanium bonds in a 10-coordinate geometry to ten equivalent germanium atoms. There is a spread of Ti–Ge bond distances ranged from 2.65 Å to 2.93 Å. Furthermore, germanium bonds in a 10-coordinate geometry to equivalent germanium and five titanium atoms. The spread of Ge–Ge bond distances is ranged from 2.66 Å to 2.92 Å.

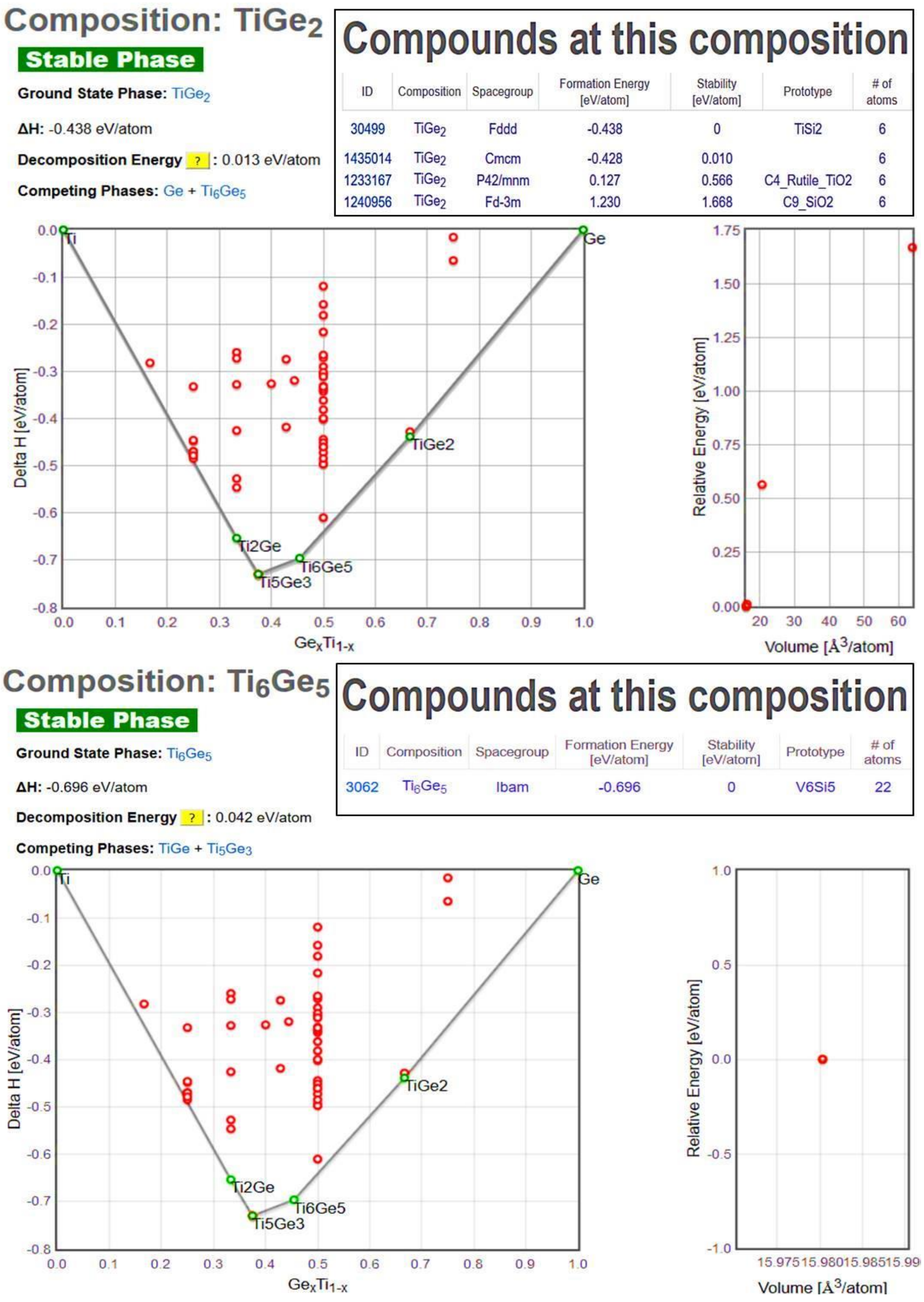

ID	Composition	Spacegroup	Formation Energy [eV/atom]	Stability [eV/atom]	Prototype	# of atoms
30499	$TiGe_2$	Fddd	-0.438	0	TiSi2	6
1435014	$TiGe_2$	Cmcm	-0.428	0.010		6
1233167	$TiGe_2$	P42/mnm	0.127	0.566	C4_Rutile_TiO2	6
1240956	$TiGe_2$	Fd-3m	1.230	1.668	C9_SiO2	6

ID	Composition	Spacegroup	Formation Energy [eV/atom]	Stability [eV/atom]	Prototype	# of atoms
3062	Ti_6Ge_5	Ibam	-0.696	0	V6Si5	22

Supplementary Figure S14: Phase stability prediction, estimated prototype and material properties computation of titanium germanide ($TiGe_2$ & Ti_6Ge_5) bonds.[6-23] In contrast with the $TiGe_2$ which includes Fddd space group in its crystallization structure, Ti_6Ge_5 has a three-dimensional structure with crystallization in the orthorhombic space group of Ibam.

a

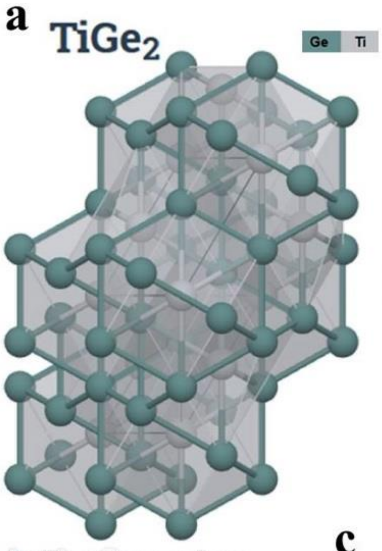

Lattice Parameters

a	6.202 Å	α	75.408°
b	5.111 Å	β	52.890°
c	5.030 Å	γ	51.702°

Volume **97.874 Å³**

b

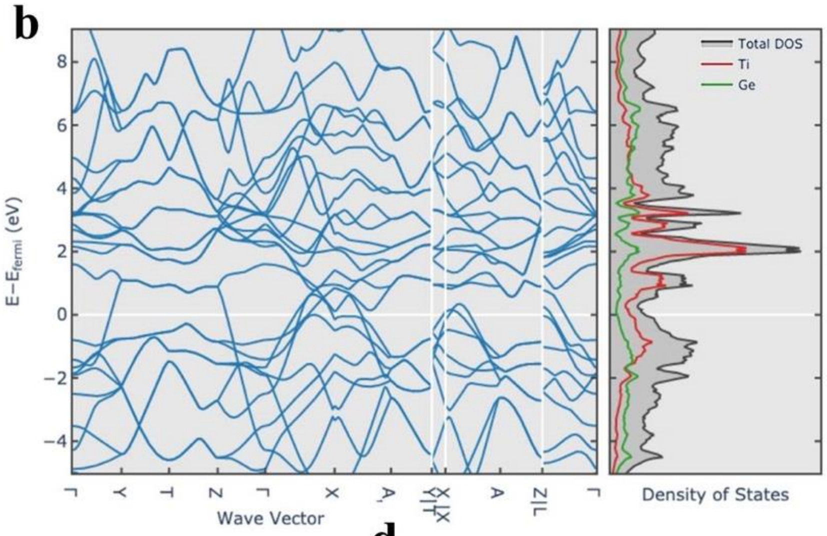

c

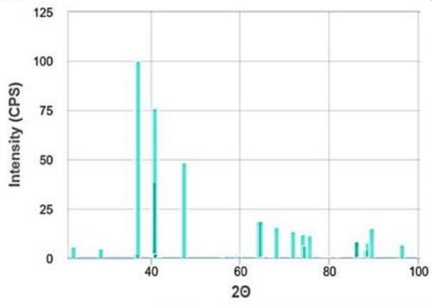

d

material	dissimilarity	E_{hull}	# of elements
$ZrSn_2$ (mp-755328)	0.0646	0.000	2
$TiSi_2$ (mp-2582)	0.1838	0.008	2
$TaGe_2$ (mp-7558)	0.2367	0.000	2
$NbGe_2$ (mp-363)	0.2555	0.000	2
Ga_2Ru (mp-1072429)	0.2775	0.000	2

e

Input: stiffness matrix (coefficients in GPa) of TiGe2

227	33	26	0	0	0
33	310	78	0	0	0
26	78	216	0	0	0
0	0	0	82	0	0
0	0	0	0	82	0
0	0	0	0	0	58

Averaging scheme	Bulk modulus	Young's modulus	Shear modulus	Poisson's ratio
Voigt	K_V = 114.11 GPa	E_V = 205.18 GPa	G_V = 85.467 GPa	ν_V = 0.20033
Reuss	K_R = 109.25 GPa	E_R = 194.26 GPa	G_R = 80.697 GPa	ν_R = 0.20364
Hill	K_H = 111.68 GPa	E_H = 199.72 GPa	G_H = 83.082 GPa	ν_H = 0.20194

Eigenvalues of the stiffness

λ_1	λ_2	λ_3
58 GPa	82 GPa	82 GPa
λ_4	λ_5	λ_6
171.05 GPa	215.5 GPa	366.45 GPa

Variations of the elastic moduli

	Young's modulus		Linear compressibility		Shear modulus		Poisson's ratio		
	E_{min}	E_{max}	β_{min}	β_{max}	G_{min}	G_{max}	ν_{min}	ν_{max}	
Value	162.11 GPa	279.34 GPa	1.955 TPa^{-1}	3.723 TPa^{-1}	58 GPa	113.1 GPa	0.079185	0.40443	Value
Anisotropy	1.723		1.9043		1.95		5.1075		Anisotropy
Axis	0.7451<br>0.6669<br>0.0000	0.0000<br>1.0000<br>0.0000	0.0000<br>1.0000<br>0.0000	1.0000<br>0.0000<br>0.0000	1.0000<br>-0.0002<br>-0.0000	0.7071<br>0.7071<br>-0.0000	0.0000<br>0.0000<br>1.0000	-0.6801<br>0.7331<br>-0.0000	Axis

f

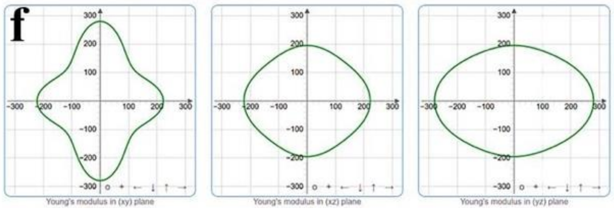

g

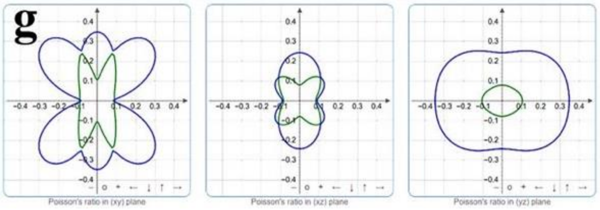

Supplementary Figure S15: Predicted crystallization and lattice parameters of $TiGe_2$ in the structure of GerMXene superlattice heterostructure. The "Materials Project" model of $TiGe_2$ in this figure provides information on **a**, the lattice space groups, **b**, energy band structure, **c**, X-

ray diffraction, **d**, dissimilarity with other similar structures, and **e-g**, elastic mechanical properties and Poisson's ratio. The results revealed crystallization pattern and detailed properties of the material. The predicted bonds (dissimilarity < 0.28) suggested the model's accuracy and validity for similar crystals such as $TaGe_2$, $NbGe_2$, $TiSi_2$ materials. This computation offers a model for other group 4 and 5 transition metals of MXenes and metalloid materials.[6-23] For total energy calculation and evaluation of compounds, the Materials Project used density functional theory as implemented in the Vienna Ab Initio Simulation Package software.

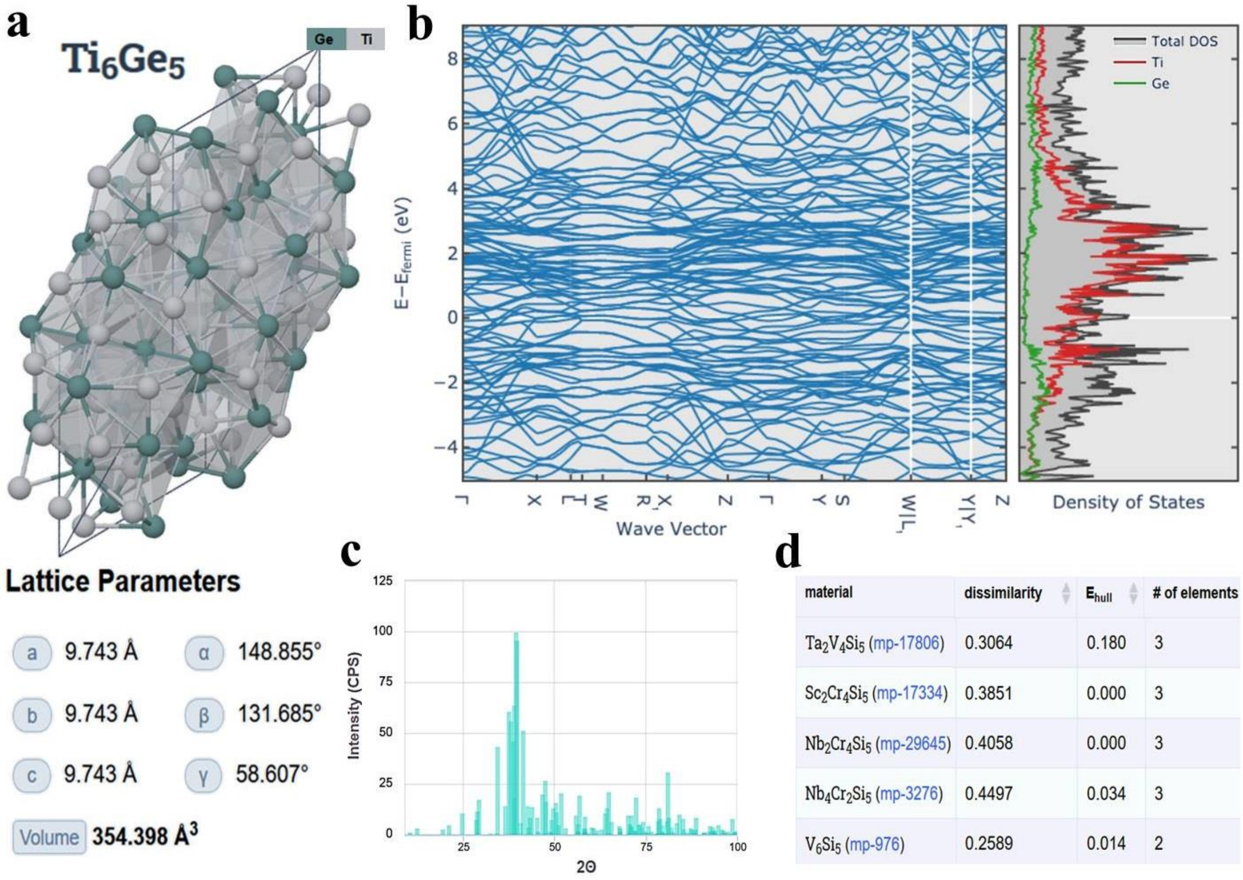

Supplementary Figure S16: The crystallization model, computed lattice parameters, band structure, density of states, X-ray diffraction and chemistry of Ti_6Ge_5. The "Materials Project" model of Ti_6Ge_5 in this figure provides information on **a**, space groups, **b**, energy band structure, **c**, X-ray diffraction, and **d**, the dissimilarity with other similar structures. Results illustrated the predicted crystallization pattern and lattice properties of the material. The predicted bonds (dissimilarity < 0.45) suggested the application of the model for similar crystals such as $Ta_2V_4Si_5$, $Nb_4Cr_2Si_5$ and V_6Si_5 materials. This computation offers a model for other group 4 and 5 transition metals of MXenes and metalloid materials.[6-23] Prediction plots suggested the relative stability of these compositions.

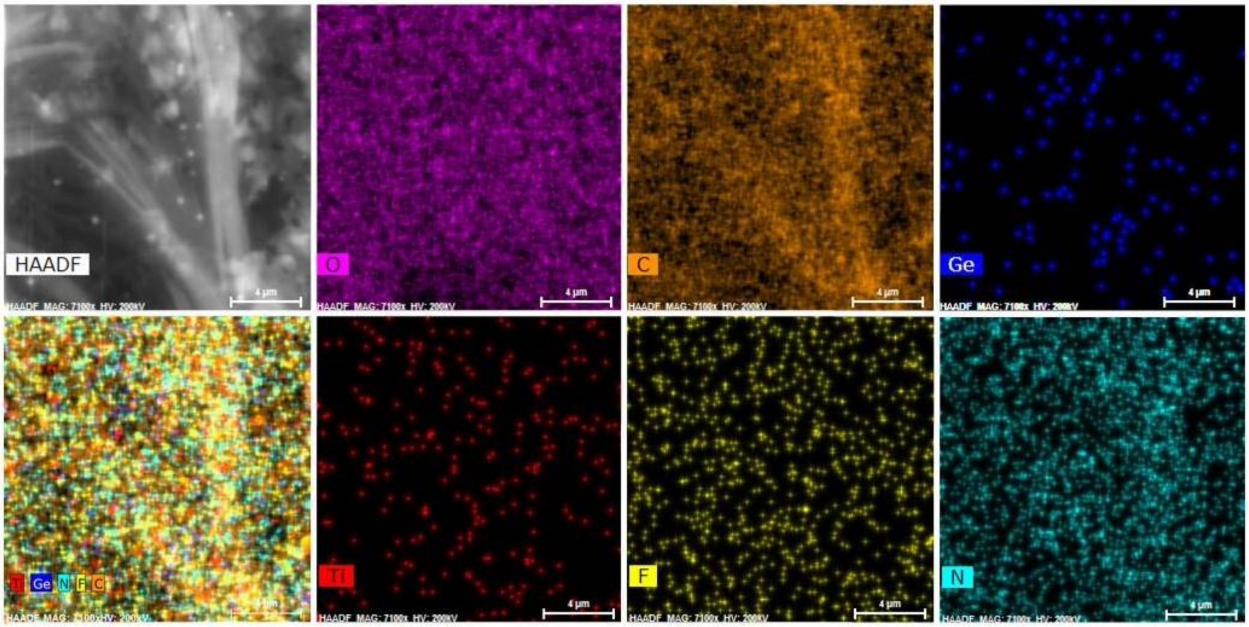

Supplementary Figure S17: Structural characterization and chemical composition of the crosslinked GerMXene-chitosan hydrogel networks. The scanning TEM micrograph and corresponding EDS elemental mapping are presented in this figure. The results show distribution of titanium (red) and germanium (blue) into the structure of GerMXene-chitosan samples.

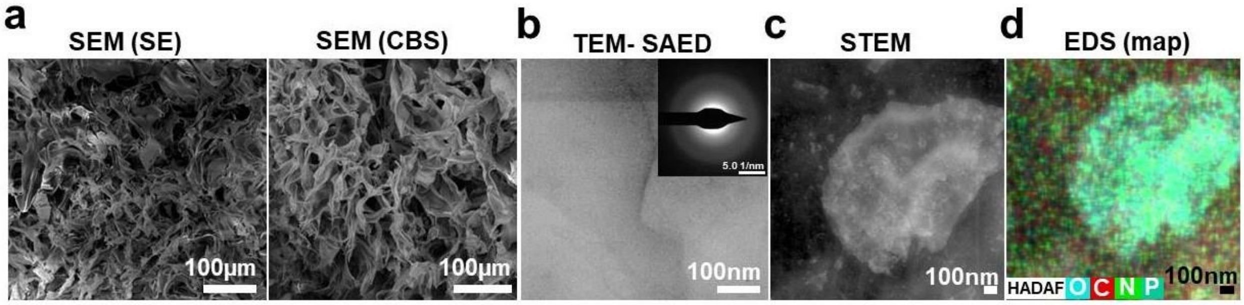

Supplementary Figure S18: Morphology and microstructural characterization of crosslinked chitosan hydrogels. a, The secondary electron detector (SE) and concentric backscatter (CBS) SEM micrographs confirm no transition-metal or metalloid traces in the structure of chitosan hydrogel. **b**, The TEM and corresponding SAED pattern further demonstrated the synthesis of pristine polymer structure with nonsignificant crystalline rings. **c** and **d**, Scanning TEM image and EDS mapping depicted the surface and elemental composition of the material.

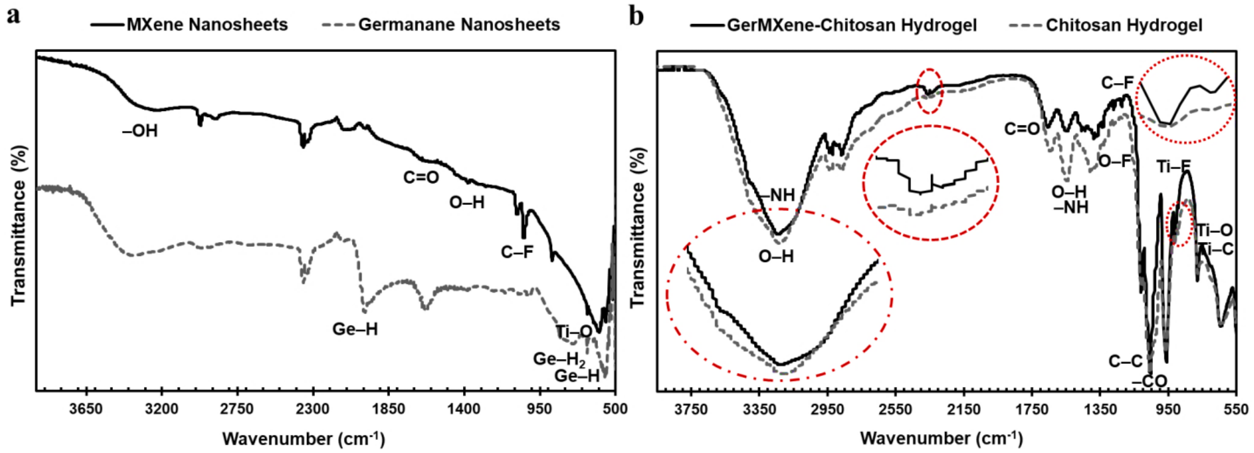

Supplementary Figure S19: Identification of chemical bonds of MXene and hydrogenated germanane (GeH) nanocrystals as well as GerMXene-chitosan and chitosan hydrogels. a, FT-IR spectra were recorded to characterize the surface functional groups of buckled 2D $Ti_3C_2T_x$ and GeH sheets. The spectrum of pristine MXene nanosheets presented the characteristics of –OH, C=O, C–O, Ti–O, Ti–C and Ti–F bonds in the structure of MXene. The Ge–H and Ge–H_2 bonds in this sample confirmed the successful synthesis of germanane crystals. **b**, The FTIR spectra of GerMXene-chitosan and chitosan revealed the presence of Ti–C, Ti–O, Ge–H, and Ge–H_2 bonds in the composites. Furthermore, a significant shift was detected in the functional groups of GerMXene-chitosan compared to the pristine chitosan samples.

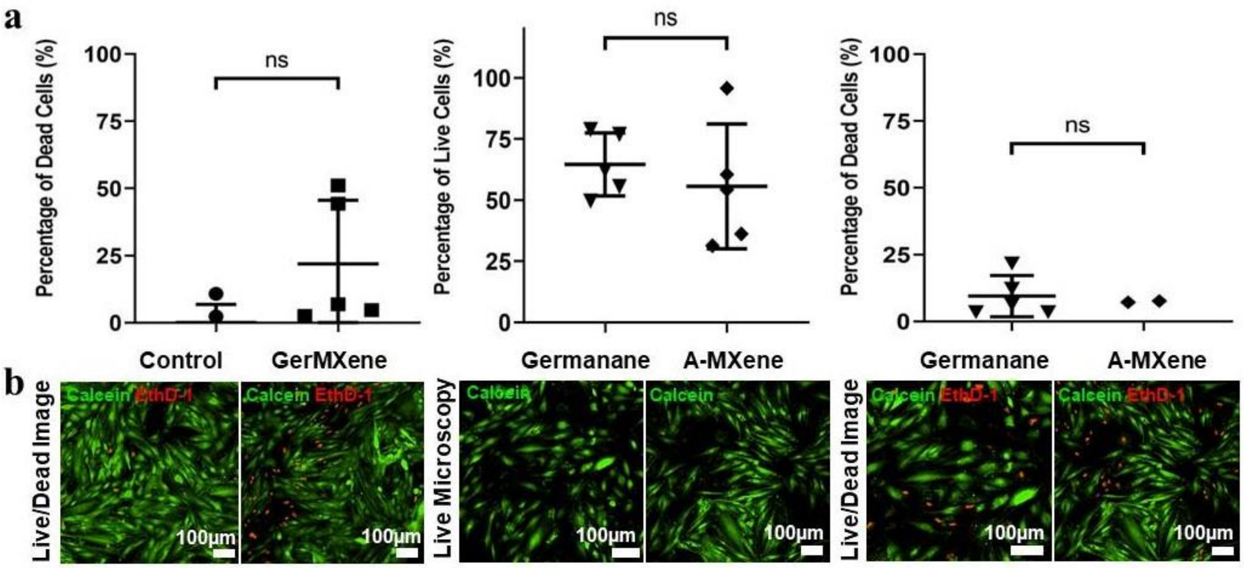

Supplementary Figure S20: Assessment of biocompatibility of aqueous colloidal GerMXene, A-MXene and 0D GeH quantum dots. a,b, Aqueous suspensions of these materials at a concentration of 100 µg mL^{-1} were co-cultured with H9C2 cells. After 24 hours of culture, the cells were stained with Calcein (to detect live cells) and EthD-1 (to detect dead cells). The viability assay and representative microscopy showed no significant cytotoxicity effect after 24 hours (n=3-5).

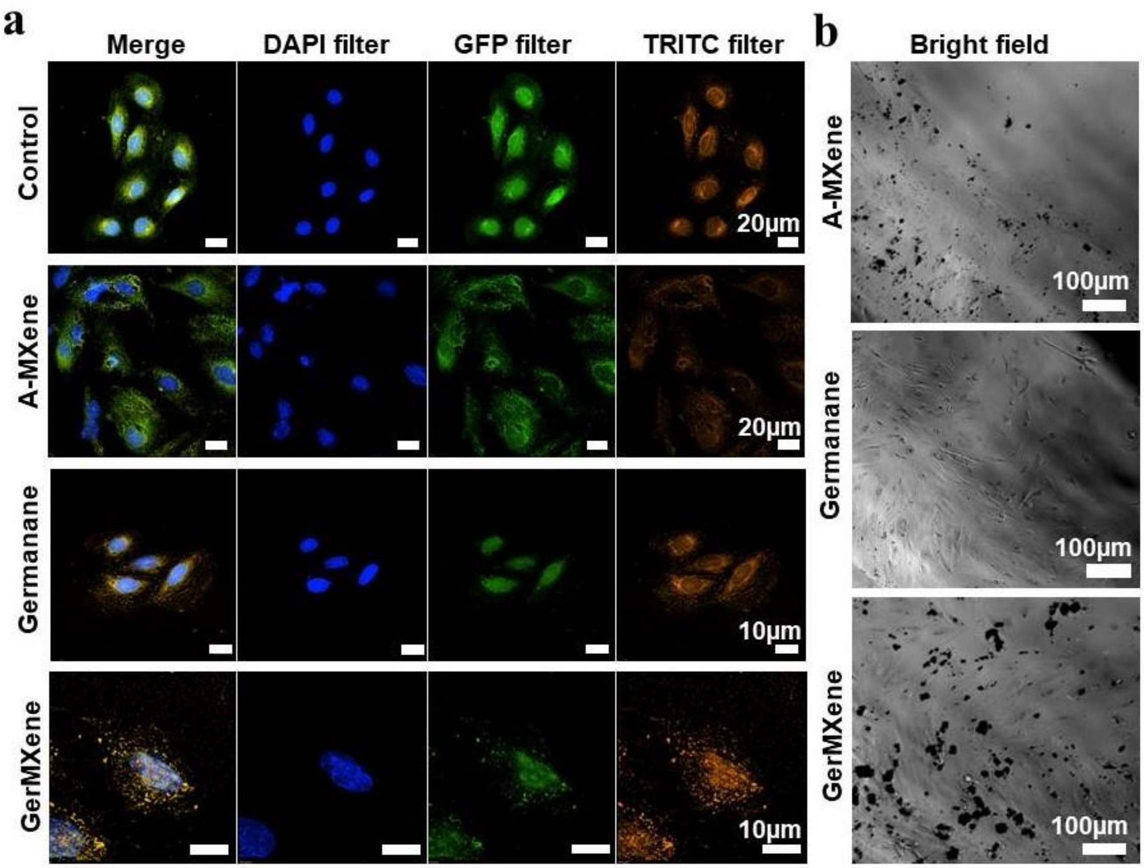

Supplementary Figure S21: Assessment of cellular uptake of aqueous GerMXene, GeH, and A-MXene. a, The tendency of cells to internalize materials was tested by co-culturing H9C2 cells with GerMXene, GeH, and A-MXene. After 24 hours of culture, the cells were stained with DAPI, fixed, and visualized by Nikon Eclipse Ti-2fluorescence microscope. The fluorescence images of H9C2 cells (DAPI stained nuclei) and materials (autofluorescence) clearly showed the localization of crystals inside the cells. The GerMXene, GeH, and A-MXene nanomaterials were readily and spontaneously uptaken by H9C2 cells without any uptake enhancing techniques. **b**, The bright-field microscopy showing interaction and internalization GerMXene, GeH, and A-MXene in H9C2 cells.

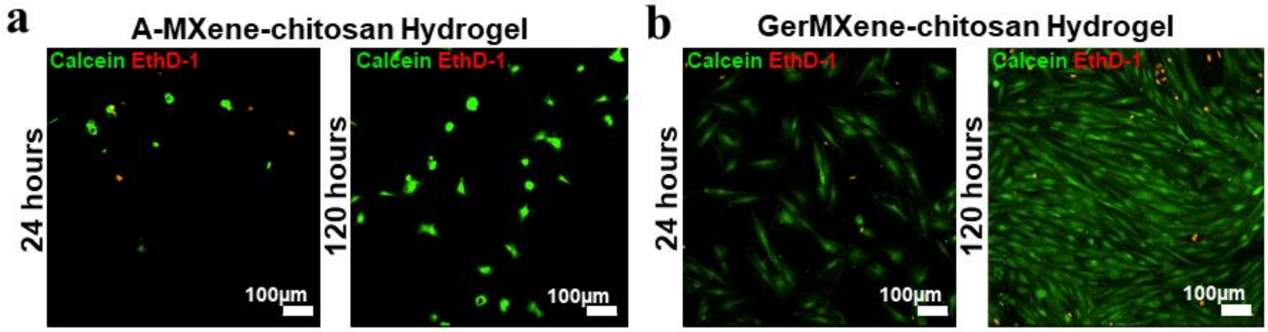

Supplementary Figure S22: Assessment of biocompatibility and bioactivity of GerMXene-chitosan compared to A-MXene-chitosan hydrogel scaffolds. The materials at a concentration of 100 µg mL^{-1} were added to polymer solutions and crosslinked at 37ºC (physiological temperature). **a-b**, A-MXene (**a**) and GerMXene (**b**) composite hydrogels were co-cultured with H9C2 cells for 24 and 120 hours. The cells were then stained with Calcein and EthD-1 to detect live and dead cells, respectively. The representative microscopic images showed a significant improvement in the attachment and survival of cells in GerMXene group after 120 hours of co-culture.

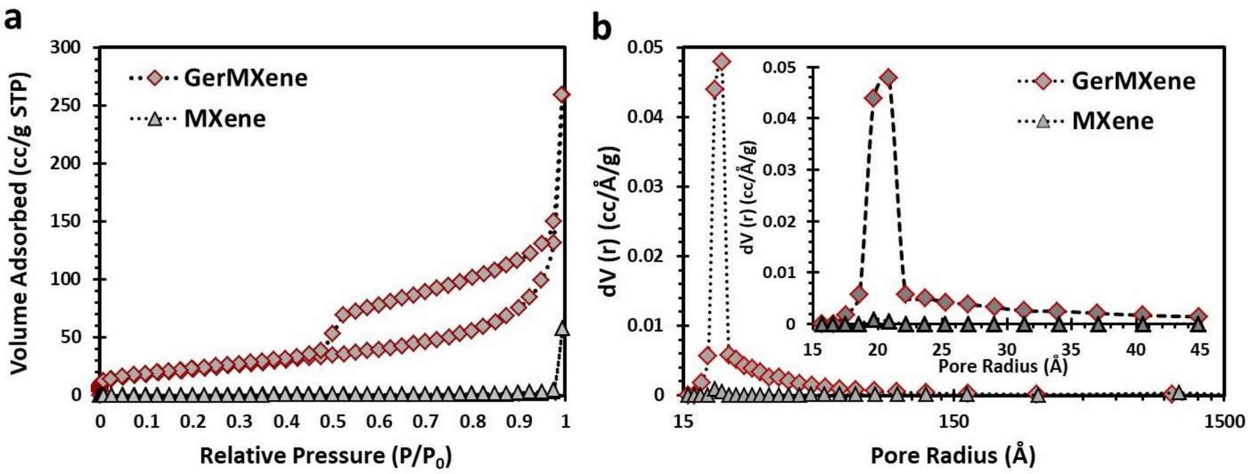

Supplementary Figure S23: Specific surface area measurements of GerMXene using Brunauer-Emmett-Teller (BET) nitrogen adsorption-desorption isotherms and Barrett-Joyner-Halenda (BJH) method. (a) N_2 adsorption-desorption isotherm curves of the $Ti_3C_2T_x$ MXene and GerMXene heterostructure. BET data demonstrate that surface area of 2D MXene nanosheets was significantly increased from 7.111 $m^2\ g^{-1}$ to 91.531 $m^2\ g^{-1}$ in GerMXene sample. **(b)** Pore size distribution of $Ti_3C_2T_x$ MXene and multi-dimensional GerMXene crystals. As shown, the average pore diameter of the MXene was decreased after conversion to GerMXene material.

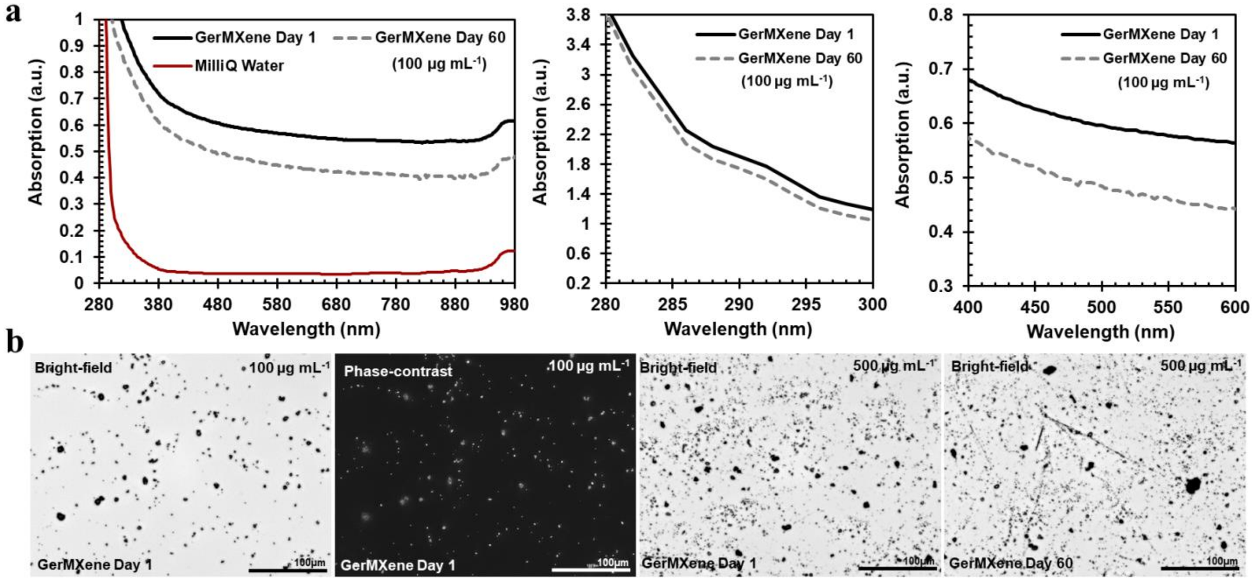

Supplementary Figure S24: UV-Vis and optical analysis of aqueous GerMXene colloids at day 1 and day 60 of synthesis. a, The UV-Vis data demonstrated no significant change in the optical absorption properties of GerMXene crystals at concentrations of 100 µg.mL^{-1}. **b**, The bright-field and phase-contrast optical microscopic images depicted uniform distribution of GerMXene particles in aqueous colloidal suspensions without significant agglomerations at tested concentrations up to 500 µg.mL^{-1}.

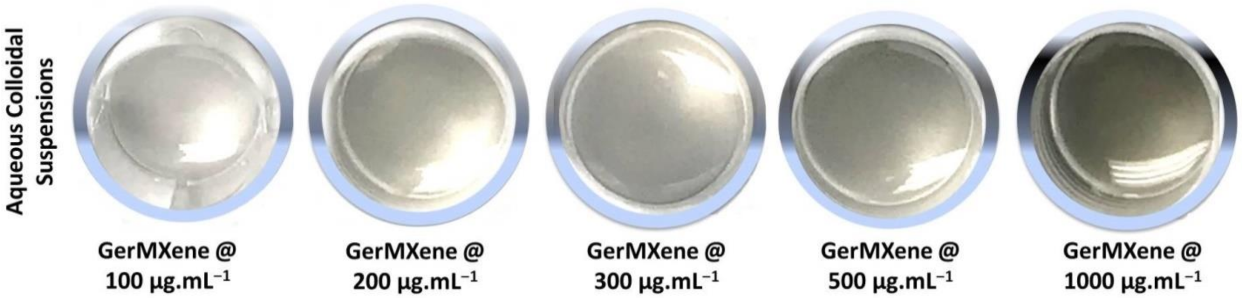

Supplementary Figure S25: Optical images of GerMXene colloids at different concentrations at room temperature. The digital images demonstrated a concentration-dependent transparency of GerMXene suspensions without any agglomeration or precipitation at room temperature at tested concentrations of up to 1000 µg.mL^{-1}.

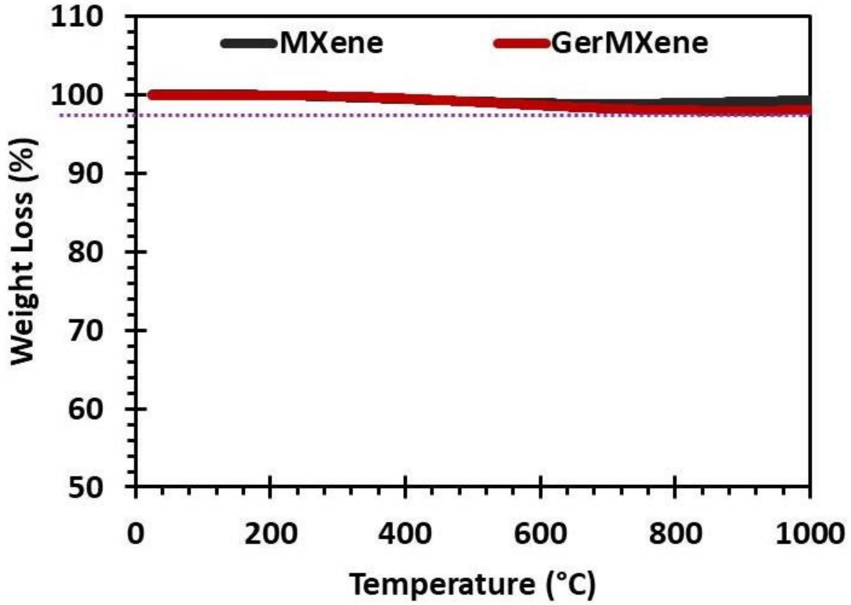

Supplementary Figure S26: Thermophysical properties and decomposition resistance of GerMXene superlattice heterostructure. Thermogravimetric analysis (TGA) of GerMXene powder is assessed and compared with $Ti_3C_2T_x$ MXene nanosheets. After annealing at a temperature up to 1000 °C under nitrogen atmosphere, the TGA analysis for GerMXene demonstrated no significant changes in the surface terminations or decomposition rate of the material. Furthermore, the TGA curves showed no significant mass loss with char residues of higher than 97%. Under nitrogen and temperature of above 600 °C conditions, the functional groups start to desorb from MXenes' surface, resulting in a minor mass loss (~ 2%). This signal is partially overlapped with the deprotonation of H_2O during measurement. The samples were pre-heated in a vacuum oven at 60 °C for 12 hours to remove water content. These data demonstrate excellent thermal stability of GerMXene at tested temperatures.

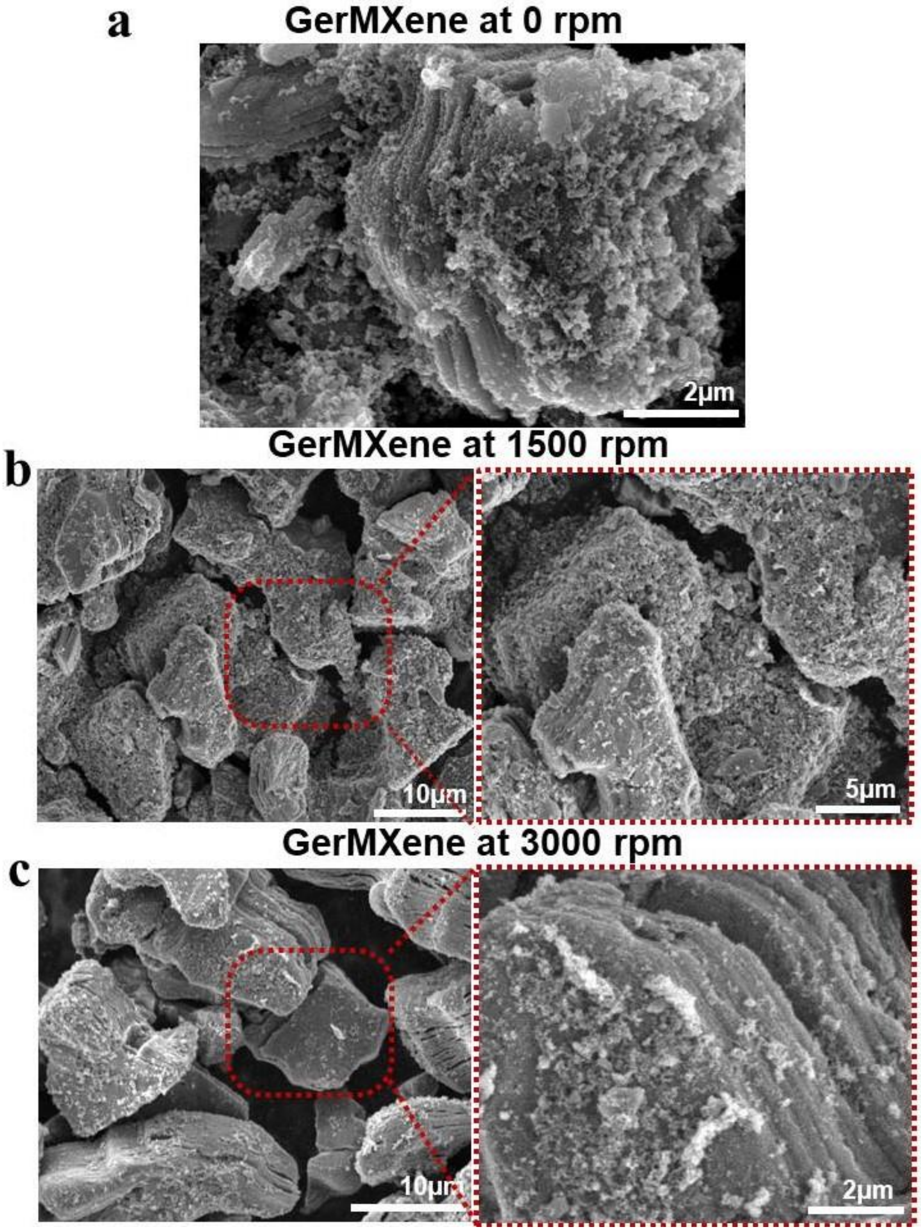

Supplementary Figure S27: Morphology of precipitated GerMXene colloids after spinning at 1500 and 3000 rpm for 15 minutes. a,b, The SEM images showed that GerMXene material at concentration of 100 μg.mL^{-1} was highly stable after centrifugation at 1500 for 15 minutes. **c**, There was no significant differences in the morphology of GerMXene suspensions by increasing the rotation speed up to 3000 rpm for 15 minutes.

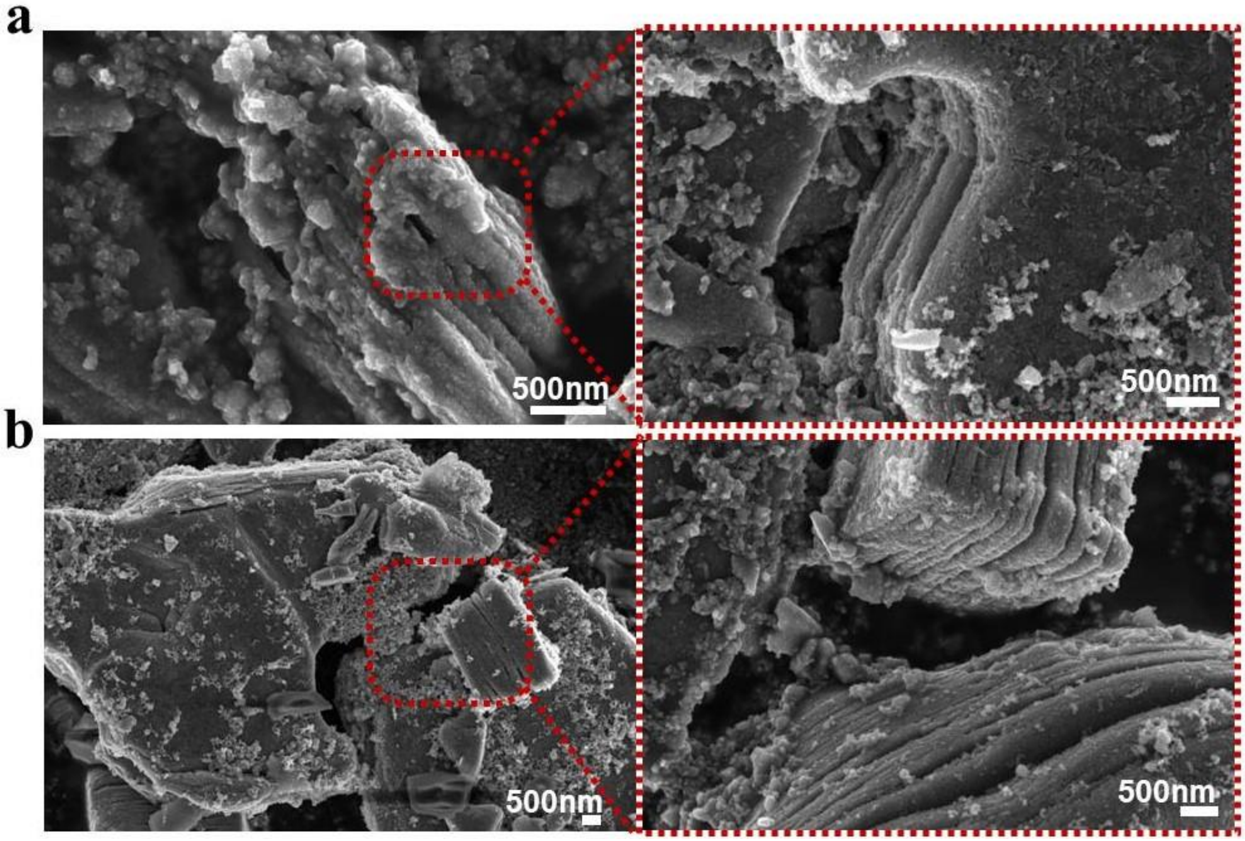

Supplementary Figure S28: Morphology characterization of GerMXene at different temperatures from 4 °C **to 70** °C**. a**, SEM images of GerMXene treated at 37 °C for 2 hours. **b**, SEM images of GerMXene heated at ~70 °C for 2 hours. Before these experiments, the material was kept overnight in the fridge at 4 °C. SEM images of GerMXene samples confirmed that storage of GerMXene at different temperatures had no significant effect on its morphology and microstructure.

Supplementary Information References

1 Huang, Shuohan, and Vadym N. Mochalin. "Hydrolysis of 2D transition-metal carbides (MXenes) in colloidal solutions." *Inorganic chemistry* **2019**, *58*, no. 3: 1958-1966.

2 Chihi, T., M. Fatmi, and M. A. Ghebouli. "Ab initio study of some fundamental properties of the M3X (M= Cr, V; X= Si, Ge) compounds." *Physica B: Condensed Matter* **2012**, *407*, no. 17: 3591-3595.

3 Thomas, O., F. M. d'Heurle, and S. Delage. "Some titanium germanium and silicon compounds: Reaction and properties." *Journal of Materials Research* **1990**, *5*, no. 7: 1453-1462.

4 Pelleg, Joshua, Reut Eliahu, Assia Barkai, and George Levi. "A note on the reactions in the Ti-Ge system." *AIP Advances* **2012**, *2*, no. 3: 032185.

5 Hallais, J, **1970**, Etude structurale des deux nouveaux composes V6 Si5 Et Ti6 Ge5. *Bulletin de la Societe Francaise de Mineralogie et de Cristallographie* (72,1949-100,1977), 93.

6 Jain, Anubhav, Shyue Ping Ong, Geoffroy Hautier, Wei Chen, William Davidson Richards, Stephen Dacek, Shreyas Cholia et al. "Commentary: The Materials Project: A materials genome approach to accelerating materials innovation." *APL materials* **2013**, *1*, no. 1: 011002.

7 Persson, Kristin, and Project, Materials. **2020**. "Materials Data on TiGe2 by Materials Project". United States. doi:10.17188/1274109. https://www.osti.gov/servlets/purl/1274109. Pub date:Thu Jul 16 00:00:00 EDT 2020.

8 Persson, Kristin, and Project, Materials. **2020**. "Materials Data on Ti6Ge5 by Materials Project". United States. doi:10.17188/1189701. https://www.osti.gov/servlets/purl/1189701. Pub date:Tue Jul 14 00:00:00 EDT 2020.

9 Gaillac, Romain, Pluton Pullumbi, and François-Xavier Coudert. "ELATE: an open-source online application for analysis and visualization of elastic tensors." *Journal of Physics*: Condensed Matter **2016**, *28*, no. 27: 275201.

10 Saal, J. E., Kirklin, S., Aykol, M., Meredig, B., and Wolverton, C. "Materials Design and Discovery with High-Throughput Density Functional Theory: *The Open Quantum Materials Database (OQMD)", JOM* **2013**, *65*, 1501-1509.

11 Kirklin, S., Saal, J.E., Meredig, B., Thompson, A., Doak, J.W., Aykol, M., Rühl, S. and Wolverton, C. "The Open Quantum Materials Database (OQMD): assessing the accuracy of DFT formation energies", *npj Computational Materials* **2015**, *1*, 15010.

12 Wallbaum, H. Ueber intermetallische Germaniumverbindungen. Naturwissenschaften, **1994**, *32*.

13 A. Jain, S.P. Ong, G. Hautier, W. Chen, W.D. Richards, S. Dacek, S. Cholia, D. Gunter, D. Skinner, G. Ceder, K.A. Persson, The Materials Project: A materials genome approach to accelerating materials innovation, *APL Materials* **2013**, 1(1), 011002. doi:10.1063/1.4812323.

14 M. de Jong, W. Chen, T. Angsten, A. Jain, R. Notestine, A. Gamst, M. Sluiter, C. K. Ande, S. van der Zwaag, J. J. Plata, C. Toher, S. Curtarolo, G. Ceder, K. A. Persson, M. Asta,

Charting the complete elastic properties of inorganic crystalline compounds Scientific Data **2015**, 2: 150009.

15 M. de Jong, W. Chen, H. Geerlings, M. Asta, K. A. Persson, *A database to enable discovery and design of piezoelectric materials, Scientific Data* **2015**, 2: 150053.

16 S. P. Ong, L. Wang, B. Kang, G. Ceder, *Li-Fe-P-O2 Phase Diagram from First Principles Calculations. Chemistry of Materials* **2008**, 20(5), 1798–1807.

17 A. Jain, G. Hautier, S. P. Ong, C. Moore, C. Fischer, K. Persson, G. Ceder. *Formation enthalpies by mixing GGA and GGA + U calculations. Physical Review B* **2011**, 84(4), 045115.

18 S. Adams and R. P. Rao. *High power lithium ion battery materials by computational design. Phys. Status Solidi A* **2011**, 208 (8), 1746-1753.

19 G. Hautier, C. Fischer, V. Ehrlacher, A. Jain, G. Ceder *Data Mined Ionic Substitutions for the Discovery of New Compounds. Inorganic chemistry* **2011**, (17), 656–663.

20 X. Qu, A. Jain, N. N. Rajput, L. Cheng, Y. Zhang, S. P. Ong, M. Brafman, E. Maginn, L. A. Curtiss, K. A. Persson, *The Electrolyte Genome project: A big data approach in battery materials discovery, Computational Materials Science* **2015**, 103, 56-57.

21 K. Mathew, C. Zheng, D. Winston, C. Chen, A. Dozier, J. J. Rehr, S. P. Ong, K. A. Persson, *High-throughput computational X-ray absorption spectroscopy, Scientific Data* **2018**, 5.

22 S. P. Ong, S. Cholia, A. Jain, M. Brafman, D. Gunter, G. Ceder, and K. A. Persson *The Materials Application Programming Interface (API): A simple, flexible and efficient API for materials data based on REpresentational State Transfer (REST) principles, Computational Materials Science* **2015**, 97, 209–215.

23 W. D. Richards, L. J. Miara, Y. Wang, J. C. Kim, G. Ceder *Interface Stability in Solid-State Batteries, Chemistry of Materials* **2016**, 28, 266–273.
